# Interplay between the plasma membrane and cell–cell adhesion maintains epithelial identity for correct polarised cell divisions

**DOI:** 10.1242/jcs.261701

**Published:** 2023-11-28

**Authors:** Manal M. Hosawi, Jiaoqi Cheng, Maria Fankhaenel, Marcin R. Przewloka, Salah Elias

**Affiliations:** ^1^ School of Biological Sciences, University of Southampton, Southampton SO17 1BJ, UK; ^2^ Institute for Life Sciences, University of Southampton, Southampton SO17 1BJ, UK

**Keywords:** Cell–cell adhesion, Chromosome segregation, Epithelial identity, Mitotic spindle, Plasma membrane remodelling, Polarised cell divisions

## Abstract

Polarised epithelial cell divisions represent a fundamental mechanism for tissue maintenance and morphogenesis. Morphological and mechanical changes in the plasma membrane influence the organisation and crosstalk of microtubules and actin at the cell cortex, thereby regulating the mitotic spindle machinery and chromosome segregation. Yet, the precise mechanisms linking plasma membrane remodelling to cell polarity and cortical cytoskeleton dynamics to ensure accurate execution of mitosis in mammalian epithelial cells remain poorly understood. Here, we manipulated the density of mammary epithelial cells in culture, which led to several mitotic defects. Perturbation of cell–cell adhesion formation impairs the dynamics of the plasma membrane, affecting the shape and size of mitotic cells and resulting in defects in mitotic progression and the generation of daughter cells with aberrant architecture. In these conditions, F- actin–astral microtubule crosstalk is impaired, leading to mitotic spindle misassembly and misorientation, which in turn contributes to chromosome mis-segregation. Mechanistically, we identify S100 Ca^2+^-binding protein A11 (S100A11) as a key membrane-associated regulator that forms a complex with E-cadherin (CDH1) and the leucine-glycine-asparagine repeat protein LGN (also known as GPSM2) to coordinate plasma membrane remodelling with E-cadherin-mediated cell adhesion and LGN-dependent mitotic spindle machinery. Thus, plasma membrane-mediated maintenance of mammalian epithelial cell identity is crucial for correct execution of polarised cell divisions, genome maintenance and safeguarding tissue integrity.

## Teaser

**Summary:** In this study, we identify a plasma membrane-mediated mechanism that maintains epithelial cell identity to ensure correct execution of polarised cell divisions, genome maintenance and safeguard tissue integrity.

## INTRODUCTION

Cell division requires equal partitioning of DNA into two daughter cells. To achieve this, each dividing cell builds a mitotic spindle that results from a spectacular rearrangement of microtubules, allowing proper alignment of chromosomes during metaphase and their equal separation during anaphase (Prosser and Pelletier, 2017). In polarised epithelia of multicellular organisms, the orientation of a correctly assembled mitotic spindle defines the position and fate of daughter cells, representing a fundamental mechanism that ensures the formation of structured and functional tissues (Lechler and Mapelli, 2021). Mitotic spindle orientation is regulated by an evolutionarily conserved ternary protein complex including the G_αi_ subunit of heterotrimeric G proteins, the leucine-glycine-asparagine repeat protein LGN (also known as GPSM2) and nuclear mitotic apparatus protein (NuMA, also known as NUMA1) (di Pietro et al., 2016). During mitosis, the G_αi_–LGN–NuMA complex localises at the cell cortex, which facilitates the interaction of NuMA with astral microtubules and the microtubule-associated minus-end motor dynein, generating pulling forces on astral microtubules that ensure correct positioning of the mitotic spindle (Carminati et al., 2016; di Pietro et al., 2016; Okumura et al., 2018; Pirovano et al., 2019). Cumulative evidence shows that the crosstalk between astral microtubules and cortical F-actin is a key mechanism for balancing the cortical forces that ensure correct mitotic spindle and chromosome dynamics, as well as mitotic progression (di Pietro et al., 2017; Dogterom and Koenderink, 2019; Fankhaenel et al., 2023; Yu et al., 2019). In mammalian epithelia, junctional proteins including E-cadherin (CDH1), afadin, ABL1, SAPCD2 and annexin A1 (ANXA1) have been shown to act as molecular landmarks instructing the polarised patterning of LGN and NuMA at the cell cortex to regulate the balance between planar and perpendicular mitotic spindle orientation (Carminati et al., 2016; Chiu et al., 2016; Fankhaenel et al., 2023; Gloerich et al., 2017; Matsumura et al., 2012). Yet, the mechanisms coordinating polarity cues with the cell cortex and the mitotic machinery in mammalian epithelial cells remain largely unknown.

During mitosis, cells transduce external cues to reorganise F-actin and microtubules at the cell cortex to control cell shape and balance intracellular tensions, thereby creating an optimal space for mitotic spindle formation and facilitating the subsequent correct alignment of the metaphase chromosomes (Lancaster et al., 2013; Leguay et al., 2022; Matthews et al., 2012, 2020; Rizzelli et al., 2020; Serres et al., 2020; Taubenberger et al., 2020). Although most of our knowledge of the mechanics of mitosis in mammalian cells comes from studies in non-polarised cells or cells grown in isolation on adhesive micropatterns (Ramkumar and Baum, 2016; Rizzelli et al., 2020; Taubenberger et al., 2020), increasing evidence shows that mitotic epithelial cells must maintain their native polarity and geometry to ensure correct mitotic spindle dynamics and chromosome segregation fidelity (Knouse et al., 2018; van Leen et al., 2020; Wang et al., 2018). Mitotic epithelial cells round up to push against tissue confinement, ensuring cell mechanics that limit spindle assembly and chromosome segregation errors (Sorce et al., 2015), while sustaining adherens junctions with their neighbouring cells to maintain epithelial integrity (Baker and Garrod, 1993; Gloerich et al., 2017; Hart et al., 2017; Reinsch and Karsenti, 1994). Reciprocally, cell rounding is influenced by tensions and mechanical forces emanating from neighbouring cells and the tissue topology, as well as the growing and remodelling tissue itself (Rizzelli et al., 2020; Taubenberger et al., 2020). Initial experiments in amphibian eggs demonstrated that the mitotic spindle aligns along the longest axis of the cell, a phenomenon referred to as Hertwig's rule (Hertwig, 1884). Subsequent in-depth studies have shown that epithelial cell anisotropy determines mitotic spindle orientation, where moderately anisotropic cells partially follow Hertwig's rule, whereas elongated cells favour division along the major axis (van Leen et al., 2020). A few planar polarity cues, such as Dishevelled proteins and VANGL2, direct mitotic spindle orientation following Hertwig's rule (Box et al., 2019; Ségalen et al., 2010). Further studies of the *Drosophila* pupal notum have shown that orthologues of LGN and NuMA (Pins and Mud, respectively) localise to tricellular junctions to act as cell shape sensors and direct planar cell division along the major axis of the cell (Bosveld et al., 2016). Consistent with this, studies of the *Xenopus* epithelium have reported that the mitotic spindle aligns to an axis of cell shape defined by the position of tricellular junctions, which requires functional cell–cell adhesion via E-cadherin protein and the localisation of LGN to tricellular junctions (Nestor-Bergmann et al., 2019). However, studies using Madin–Darby canine kidney (MDCK) cells have shown that E-cadherin and cortical LGN align epithelial cell divisions with tissue tension independently of cell shape, and that the localisation of E-cadherin at the plasma membrane is key to the patterning of LGN at the cell cortex (Gloerich et al., 2017; Hart et al., 2017). The precise molecular mechanisms allowing E-cadherin-mediated adhesion to transduce external cues to ensure correct execution of polarised cell divisions remain not well understood in mammalian epithelial cells.

Mitotic cells undergo dynamic changes in their volume and surface topology that are driven by remodelling in the plasma membrane, which responds to mechanical stresses and connects with the cortex to fine-tune intracellular tensions that control the orientation, progression and outcome of cell division (Carlton et al., 2020; Ramkumar and Baum, 2016). Depletion of plasma membrane proteins such as the G-protein-coupled receptors can inhibit cell division (Zhang et al., 2012; Zhang and Eggert, 2013). Other studies have reported differences in the cell surface proteome between interphase and metaphase cells (Özlü et al., 2015). In single HeLa cells, elongation of the plasma membrane is coordinated with cortical localisation of dynein to centre the mitotic spindle in anaphase and achieve symmetric cell division (Kiyomitsu and Cheeseman, 2013). In these cells, polar plasma membrane blebbing stabilises cell shape by relieving actomyosin cortical tensions, ensuring the stability of cleavage furrow positioning for correct progression of cytokinesis (Sedzinski et al., 2011). In polarised epithelial cells, on the other hand, decreases in intracellular tensions rely on traction forces from neighbouring cells in addition to those emanating from the extracellular matrix (Uroz et al., 2019; Uroz et al., 2018), further highlighting the functional requirement of cell–cell signalling for correct execution of polarised cell divisions. However, it remains unknown how the dynamic progression and outcomes of mitosis are coordinated with plasma membrane remodelling and cell–cell adhesion.

Here, we exploit a simple monolayer culture system combined with fluorescence time-lapse and confocal imaging to experimentally manipulate the density of mammary epithelial cells and examine how perturbation of cell–cell adhesion formation influences the orientation, mechanics and outcome of cell division. We show that cells grown at low density lose their polarised epithelial identity and display aberrant dynamics of the plasma membrane during mitosis, which affects the size and shape of mitotic cells and results in daughter cells with architectural defects. In these non-polarised conditions, cortical actin organisation and its interaction with astral microtubules are impaired. Consequently, cells fail to correctly align the mitotic spindle and chromosomes, leading to delayed mitotic progression and cytokinesis defects. In this experimental context, we investigated the function of the membrane-associated protein S100 Ca^2+^-binding protein A11 (S100A11), which we have recently identified in a proteomic screen of mitotic mammary epithelial cells (Fankhaenel et al., 2023). S100A11 associates with the plasma membrane in a Ca^2+^-dependent manner and has been shown to regulate plasma membrane repair in migrating cells by facilitating dynamic organisation of F-actin and remodelling of the plasma membrane (Jaiswal et al., 2014; Mohammed et al., 2023 preprint). S100A11 is enriched in pseudopodia of metastatic cancer cells and is required for the formation of actin-dependent pseudopodia protrusions and tumour cell migration (Shankar et al., 2010). Furthermore, S100A11 has been identified as an interactor of E-cadherin at adherens junctions (Guo et al., 2014). In mitotic mammary epithelial cells, we have recently shown that localisation of S100A11 and its direct partner ANXA1 at the plasma membrane is required for polarised accumulation of LGN at the lateral cortex to promote planar cell division (Fankhaenel et al., 2023). However, the precise function of S100A11 in mitosis has not been characterised yet. In the present experiments, we demonstrate that S100A11 is required for proper plasma membrane remodelling to ensure faithful segregation of chromosomes and the generation of equal-sized daughter cells. We show that S100A11 forms a complex with E-cadherin and LGN to instruct correct E-cadherin-mediated adhesion and lateral patterning of the LGN spindle orientation machinery, thereby ensuring planar cell division. S100A11 depletion phenocopies the mitotic defects observed in non-polarised cells and alters epithelial integrity. Collectively, our experiments shed new light on the importance of epithelial identity maintenance for correct dynamics, mechanics and outcome of polarised cell divisions, and our results suggest that S100A11-mediated plasma membrane remodelling coordinates mechanochemical crosstalk between cell–cell adhesion, the cell cortex and the mitotic spindle machinery in mammary epithelial cells.

## RESULTS

### Epithelial cell density-dependent plasma membrane remodelling influences mitosis progression and outcome

**Fig. 1.**
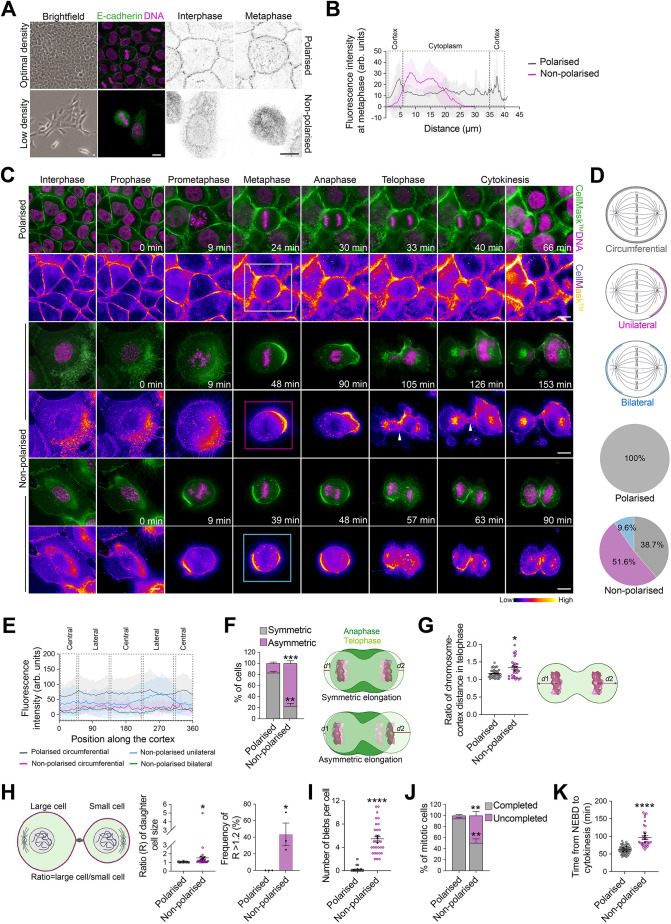
**Perturbation of cell–cell adhesion formation results in asymmetric plasma membrane elongation and defects in mitosis progression and outcome.** (A) Brightfield and confocal images of representative polarised and non-polarised MCF-10A cells stained for E-cadherin (green) and counterstained with Hoechst 33342 (DNA, magenta). Greyscale images show E-cadherin. (B) Mean cortical and cytoplasmic fluorescence intensity profiles of E-cadherin in polarised and non-polarised metaphase cells (polarized, *n*=26 cells; non-polarised, *n*=24 cells). (C) Time-lapse images of representative polarised and non-polarised cells. The plasma membrane was labelled with CellMask (green) and DNA was labelled with Hoechst 33342 (magenta), 10 min and 30 min before acquisition, respectively. Images showing CellMask intensity using a fire lookup table are presented beneath each time course. Arrowheads indicate accumulation of vesicles at the cleavage furrow in non-polarised cells. Boxes indicate metaphase cells showing the localisation phenotypes of CellMask quantified in D: circumferential (grey), unilateral (pink) and bilateral (blue). (D) Percentage of cells with the indicated CellMask labelling distribution at the cell surface during metaphase for polarised and non-polarised cells (polarized, *n*=30 cells; non-polarised, *n*=30 cells). (E) Mean cortical fluorescence intensity profiles of CellMask from polarised and non-polarised cells (polarized, *n*=30 cells; non-polarised, *n*=30 cells). Position is shown as angle (°). (F) Percentage of cells with symmetric and asymmetric plasma membrane elongation (polarized, *n*=34 cells; non-polarised, *n*=32 cells). Distances (*d*1 and *d*2) were measured as shown in the illustration. Unpaired two-tailed Student's *t*-test: symmetric, ***P=0.0003; asymmetric, **P=0.0012. (G) Ratio of chromosome to cortex distance (*d*1/*d*2) in telophase cells (polarised, *n*=37 cells; non-polarised, *n*=30 cells). Unpaired two-tailed Student's *t*-test: *P=0.014. Distances were measured as shown in the illustration. (H) Middle: ratio (R) of daughter cell size. Unpaired two-tailed Student's *t*-test, *P=0.018. Right: frequency of R>1.2. Unpaired two-tailed Student's *t*-test, *P=0.0326. Polarised, *n*=35 cells; non-polarised, *n*=30 cells. Ratio was measured as shown in the illustration (left). (I) Number of polar blebs per cell at anaphase-to-telophase transition (polarised, *n*=30 cells; non-polarised, *n*=30 cells). Unpaired two-tailed Student's *t*-test, ****P<0.0001. (J) Percentage of cells with completed and uncompleted mitosis (polarised, *n*=63 cells; non-polarised, *n*=67 cells). Unpaired two-tailed Student's *t*-test, **P=0.0013. (K) Time from nuclear envelope breakdown (NEBD) to cytokinesis (polarised, *n*=58 cells; non-polarised, *n*=34 cells). Unpaired two-tailed Student's *t*-test, ****P<0.0001. All data are presented as mean±s.e.m. from three or four independent experiments. In G–I and K, coloured data points represent mitotic cells, black data points represent independent experiments. Arb. units, arbitrary units. Scale bars: 10 µm. Source data are provided in Table S1.

To examine the functional requirement of cell–cell adhesion for correct plasma membrane remodelling during cell division, we used human MCF-10A mammary epithelial cells cultured at optimal or low density (Fig. 1A). Characterisation of MCF-10A monolayers 72 h after plating revealed that cells cultured at optimal density established E-cadherin adherens junctions, whereas cells cultured at low density lost their polarised epithelial identity and failed to form correct cell–cell adhesions, with E-cadherin abnormally accumulating in the cytoplasm both in interphase and metaphase, which allowed us to define optimal-density MCF-10A cells as polarised and low-density MCF-10A cells as non-polarised (Fig. 1A,B). Next, we performed live imaging of polarised and non-polarised MCF-10A cells, in which we labelled the plasma membrane with CellMask and DNA with Hoechst 33342. Consistent with previous studies (Chaigne et al., 2021), CellMask displayed a homogeneous, circumferential distribution at the cell surface of polarised mitotic cells, which generated equal-sized daughter cells at cytokinesis (Fig. 1C–E; Movie 1). In non-polarised mitotic cells, however, the plasma membrane had aberrant dynamics, with ∼52% and ∼39% of cells displaying a unilateral and bilateral accumulation of CellMask at the cell surface, respectively (Fig. 1C–E; Movies 2 and 3). Perturbation of cell–cell adhesion formation affected the size and shape of non-polarised cells, which displayed a mesenchymal behaviour associated with polar, asymmetric elongation of the plasma membrane during anaphase in ∼77% of mitotic cells (Fig. 1C,F). In these conditions, sister chromatids were off-centred from anaphase until telophase, which affected the position of the cleavage furrow and generated unequal-sized daughter cells at cytokinesis in ∼44% of non-polarised mitotic cells (Fig. 1G,H), which is consistent with previous findings in HeLa cells (Kiyomitsu and Cheeseman, 2013). During anaphase-to-telophase transition, the plasma membrane of non-polarised cells formed blebs in the polar area opposing the expanding cell cortex, but this failed to recentre the sister chromatids or stabilise the cleavage furrow (Fig. 1C,I), which disagrees with previous studies showing that polar membrane blebbing influences the position and stability of the mitotic spindle and cleavage furrow (Kiyomitsu and Cheeseman, 2013; Sedzinski et al., 2011). Interestingly, a close examination of the CellMask labelling in non-polarised cells revealed an accumulation of cytoplasmic vesicles, some of which localised to the cleavage furrow (Fig. 1C). Thus, asymmetric membrane elongation might involve both remodelling and *de novo* incorporation of membrane components into the cleavage furrow. We also observed significant defects in the dynamics of cell division in a vast majority of non-polarised cells as compared to polarised cells, with the proportion of cells that completed mitosis decreasing significantly in low-density cell cultures (non-polarised, ∼50%; polarised, ∼94%; Fig. 1J). In non-polarised cells that completed their division, the duration of mitosis was extended (Fig. 1C), as revealed by an increased mean transition time from nuclear envelope breakdown to cytokinesis (non-polarised, ∼98 min; polarised, ∼62 min) (Fig. 1K), which is consistent with a recent study in MDCK cells showing that epithelial density influences progression of the cell cycle (Donker et al., 2022). Taken together, these data suggest that cell–cell adhesion and plasma membrane remodelling are coordinated to maintain polarised epithelial identity, thereby ensuring correct mechanics and progression of mitosis as well as symmetric cytokinesis.

### Epithelial cell density-dependent mitotic spindle dynamics and chromosome segregation fidelity

**Fig. 2.**
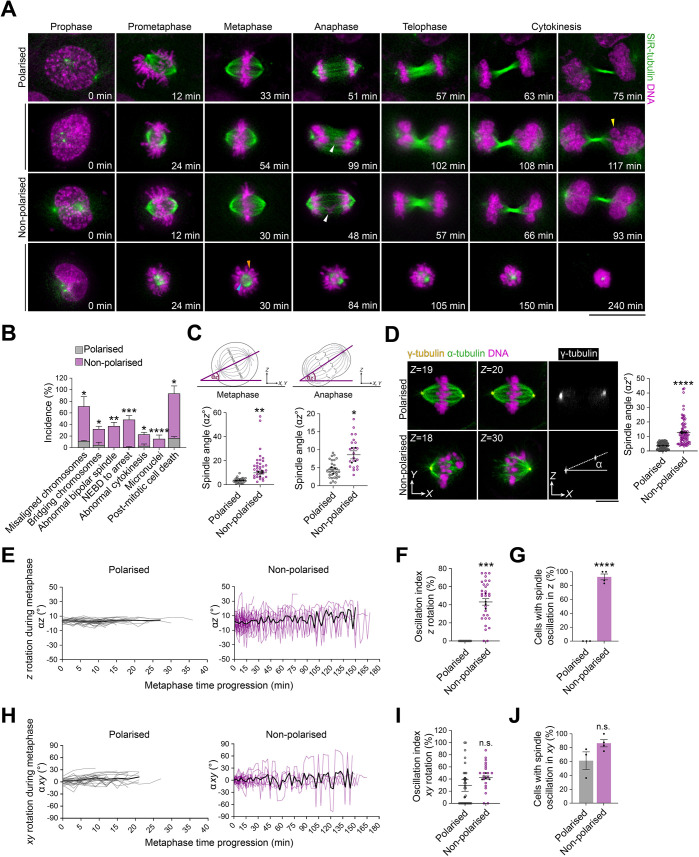
**Perturbation of cell–cell adhesion formation impairs mitotic spindle dynamics and chromosome segregation.** (A) Time-lapse images of representative polarised and non-polarised MCF-10A cells. Microtubules were labelled with SiR-tubulin (green) and DNA was labelled with Hoechst 33342 (magenta), 3 h and 30 min before acquisition, respectively. The coloured arowheads indicate the phenotypes listed in B (white: bridging chromosomes; yellow: micronuclei; orange: misaligned chromosomes; cyan: abnormal bipolar spindle). Scale bar: 10 µm. (B) Percentage of cells with the indicated defects in mitotic spindle assembly, chromosome alignment and segregation, and mitosis progression and outcome (NEBD, nuclear envelope breakdown) (polarised, *n*=61 cells; non-polarised, *n*=73 cells). Unpaired two-tailed Student's *t*-test: misaligned chromosomes, *P=0.043; bridging chromosomes, *P=0.029; abnormal bipolar spindle, **P=0.0087; NEBD-to-arrest, ***P=0.0006; abnormal cytokinesis, *P=0.0298; micronuclei, ****P<0.0001; and post-mitotic cell death, *P=0.0462. (C) Mitotic spindle angles α*z* from time-lapse images in polarised and non-polarised cells at metaphase (left) (polarised, *n*=30 cells; non-polarised, *n*=30 cells) and anaphase (right) (polarised, *n*=30 cells; non-polarised, *n*=21 cells). Unpaired two-tailed Student's *t*-test: metaphase, **P=0.0034; anaphase, *P=0.0316. (D) Left: confocal images of representative polarised and non-polarised cells stained for α-tubulin (green) and γ-tubulin (yellow), and counterstained with Hoechst 33342 (DNA, magenta). *Z* section numbers are shown for *xy* views. Scale bar: 10 µm. Right: mitotic spindle angle α*z* in fixed polarised and non-polarised cells during metaphase (polarised, *n*=103 cells; non-polarised, *n*=74 cells). Unpaired two-tailed Student's *t*-test, ****P<0.0001. (E) Dynamics of *z* orientation (α*z*) in polarised cells (left) and non-polarised cells (right) (polarised, *n*=30 cells; non-polarised, *n*=30 cells). Black lines represent the mean spindle angles. (F) Oscillation index in polarised and non-polarised cells in the *z* axis (polarised, *n*=30 cells; non-polarised, *n*=30 cells). Unpaired two-tailed Student's *t*-test, ***P=0.0002. (G) Percentage of cells with spindle oscillation in the *z* axis (polarised, *n*=30 cells; non-polarised, *n*=30 cells). Unpaired two-tailed Student's *t*-test, ****P<0.0001. (H) Dynamics of *xy* orientation (α*xy*) in polarised cells (left) and non-polarised cells (right) (polarised, *n*=30 cells; non-polarised, *n*=28 cells). Black lines represent the mean spindle angles. (I) Oscillation index in polarised and non-polarised cells in the *xy* axis (polarised, *n*=30 cells; non-polarised, *n*=30 cells). Unpaired two-tailed Student's *t*-test, *P*=0.203. (J) Percentage of cells with spindle oscillation in the *xy* axis (polarised, *n*=30 cells; non-polarised, *n*=29 cells). Unpaired two-tailed Student's *t*-test, *P*=0.0968. All data are presented as mean±s.e.m. from three or four independent experiments. In C, D, F and I, coloured data points represent mitotic cells, black data points represent independent experiments. n.s., not significant. Source data are provided in Table S1.

The results above strongly suggest that epithelial cell density influences the assembly and alignment of the mitotic spindle, which in turn ensures correct chromosome dynamics and segregation. To test this hypothesis, we performed live imaging of polarised and non-polarised MCF-10A cells, in which we labelled microtubules with SiR-tubulin (Lukinavičius et al., 2014) and DNA with Hoechst 33342 (Fig. 2A). We identified several mitotic defects in non-polarised cells (Fig. 2A,B; Movies 4–7). We observed significant defects in chromosome alignment and segregation in ∼60% and ∼27% of non-polarised cells, respectively, which led to a significant increase in the proportion of daughter cells inheriting micronuclei at cytokinesis (non-polarised, ∼15%; polarised, 0%; Fig. 2A,B; Movie 5). The morphology of the mitotic spindle was also affected, with ∼35% of non-polarised cells displaying abnormal bipolar spindles (Fig. 2A,B; Movie 7). During metaphase, polarised cells aligned their mitotic spindle parallel to the substratum plane (spindle angle ∼4°), whereas non-polarised cells displayed spindle orientation defects (spindle angle ∼10°), which persisted during anaphase (non-polarised, spindle angle ∼9°; polarised, spindle angle ∼4°; Fig. 2C). We further confirmed these observations using immunofluorescence and confocal imaging (Fig. 2D). Our live imaging experiments also revealed that ∼93% of non-polarised cells displayed excessive oscillations of the mitotic spindle relative to the *z* axis between successive time frames during metaphase, whereas the spindle in polarised cells did not display notable oscillatory *z* rotations and remained in a planar position (Fig. 2E–G). There was also an increase in oscillatory rotations of the mitotic spindle in the *xy* plane in non-polarised cells as compared to polarised cells, but the difference was not significant (Fig. 2H–J). Nonetheless, whereas all investigated polarised cells aligned their mitotic spindle in accordance with Hertwig's rule in the *xy* plane, only ∼28% of non-polarised cells followed this rule (Fig. S1A,B). Thus, cell–cell adhesion-mediated polarisation of mammary epithelial cells ensures correct mitotic spindle assembly and dynamic orientation, and in turn, faithful chromosome segregation.

### Epithelial cell density-dependent reorganisation of cortical actin during mitosis

**Fig. 3.**
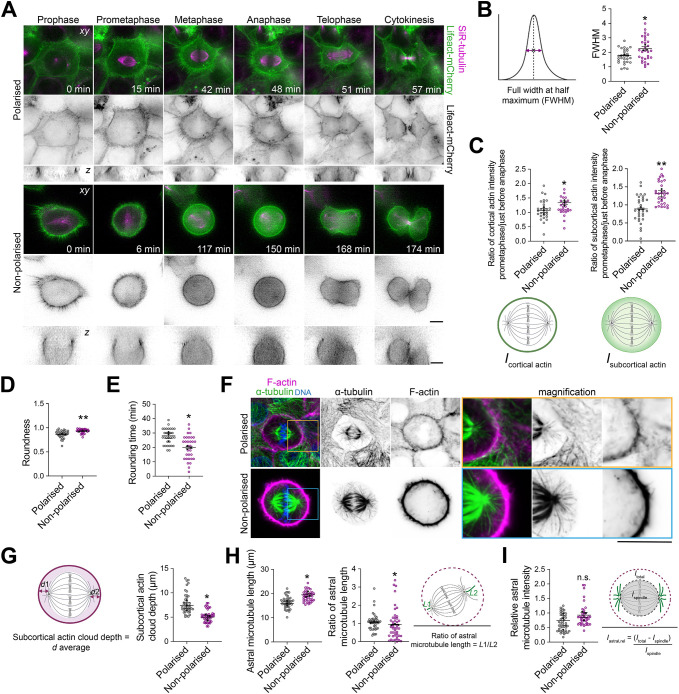
**Perturbation of cell–cell adhesion formation impairs F-actin and astral microtubule organisation and crosstalk.** (A) Time-lapse images of representative clonal polarised and non-polarised MCF-10A cells stably expressing Lifeact–mCherry (green). Microtubules were labelled with SiR-tubulin (magenta) 3 h before imaging. Scale bars: 10 µm. (B) Full width at half maximum (FWHM; illustrated in the schematic) for measurement of cortical actin thickness (polarised, *n*=29 cells; non-polarised, *n*=30 cells). Unpaired two-tailed Student's *t*-test, *P=0.0169. (C) Ratio of fluorescence intensity of cortical (left; *I*_cortical actin_) and subcortical (right; *I*_subcortical actin_) actin between prometaphase and the last frame of metaphase. Cortical: polarised, *n*=30 cells; non-polarised, *n*=29 cells. Subcortical: polarised, *n*=28 cells; non-polarised, *n*=25 cells. Unpaired two-tailed Student's *t*-test: cortical, *P=0.044; subcortical, **P=0.010. (D) Roundness of metaphase cells (polarised, *n*=33 cells; non-polarised, *n*=29 cells). Unpaired two-tailed Student's *t*-test, **P=0.0035. (E) Rounding time for polarised and non-polarised cells (polarised, *n*=31 cells; non-polarised, *n*=31 cells). Unpaired two-tailed Student's *t*-test, *P=0.0159. (F) Confocal images of representative polarised and non-polarised cells stained for F-actin (magenta) and α-tubulin (green), and counterstained with Hoechst 33342 (DNA, blue). Boxes indicate regions shown in magnified views. Scale bar: 10 µm. (G) Average subcortical actin cloud depth (*d*) in polarised and non-polarised cells (polarised, *n*=41 cells; non-polarised, *n*=43 cells). Unpaired two-tailed Student's *t*-test, *P=0.029. Depths *d*1 and *d*2 were measured as shown in the illustration, and the average value was taken. (H) Left: astral microtubule length (polarised, *n*=42 cells; non-polarised, *n*=39 cells). Middle: ratio of astral microtubule length (polarised, *n*=42 cells; non-polarised, *n*=45 cells). Unpaired two-tailed Student's *t*-test: astral microtubule length, *P=0172; ratio of astral microtubule length, *P=0176. Right: lengths *L*1 and *L*2 were measured as shown in the illustration. (I) Relative fluorescence intensities of astral microtubules (*I*_astral, rel_) in polarised and non-polarised cells (polarised, *n*=43 cells; non-polarised, *n*=39 cells). Unpaired two-tailed Student's *t*-test, *P*=0.37. The total cellular fluorescence intensity (*I*_total_) and spindle fluorescence intensity (*I*_spindle_) were measured as shown in the illustration. All data are presented as mean±s.e.m. from three independent experiments. Coloured data points represent mitotic cells, black data points represent independent experiments. n.s., not significant. Source data are provided in Table S1.

Dynamic reorganisation of actin cytoskeleton into a uniform, contractile cortex at the cell surface at mitotic entry ensures proper assembly and orientation of the mitotic spindle by polarising cortical force-generating proteins that pull on astral microtubules (Carminati et al., 2016; Fankhaenel et al., 2023; Yu et al., 2019). This cortical actin network is important for accurate control of cell rounding, which peaks during metaphase (Rizzelli et al., 2020; Taubenberger et al., 2020). In epithelial cells, mitotic rounding defects result in abnormal spindle assembly and orientation, division asymmetries, as well as delayed mitotic progression (Chanet et al., 2017; Lancaster et al., 2013). To investigate how perturbation of cell–cell adhesion formation influences cortical actin reorganisation during mitosis, we performed live imaging in MCF-10A cells stably expressing Lifeact–mCherry and labelled with SiR-tubulin (Fig. 3A; Movies 8 and 9). We measured cortical thickness in metaphase, which is a key readout of cortical actin architecture (Serres et al., 2020), and found that perturbation of cell–cell adhesion formation led to an increase in cortex thickness (Fig. 3B). F-actin fluorescence intensity also increased in both the cortical and subcortical regions of non-polarised metaphase cells (Fig. 3C). Consequently, non-polarised cells were rounder, and the rounding-up process was faster as compared to that in polarised cells (non-polarised, ∼20 min; polarised, ∼28 min; Fig. 3D,E). We confirmed these observations using immunofluorescence and confocal imaging, which also revealed a decrease in the depth of the subcortical actin cloud in non-polarised cells (non-polarised, ∼5 µm; polarised, ∼8 µm; Fig. 3F,G; Fig. S2A–C). The subcortical actin cloud has been shown to mediate cortical forces and to concentrate force-generating proteins at the retraction fibres, which act on centrosome dynamics and pull on astral microtubules to orient the mitotic spindle (Kwon et al., 2015). We found that subcortical actin cloud thinning was associated with impaired astral microtubule length in non-polarised cells (non-polarised, ∼19 nm; polarised, ∼16 nm), whereas the relative intensity of the astral microtubules remained unaffected, suggesting their stabilisation (Fig. 3H,I). Finally, the length of the mitotic spindle and the fluorescence intensity of spindle microtubules were increased in non-polarised cells as compared to polarised cells (Fig. S2D,E). Thus, cell–cell adhesion-mediated polarised epithelial cell identity defines the dynamic reorganisation of the actin cortex and its interaction with astral microtubules to ensure correct mitotic spindle dynamics and mitotic cell rounding.

### S100A11 coordinates plasma membrane remodelling with cell–cell adhesion and cortical cytoskeleton organisation during mitosis

**Fig. 4.**
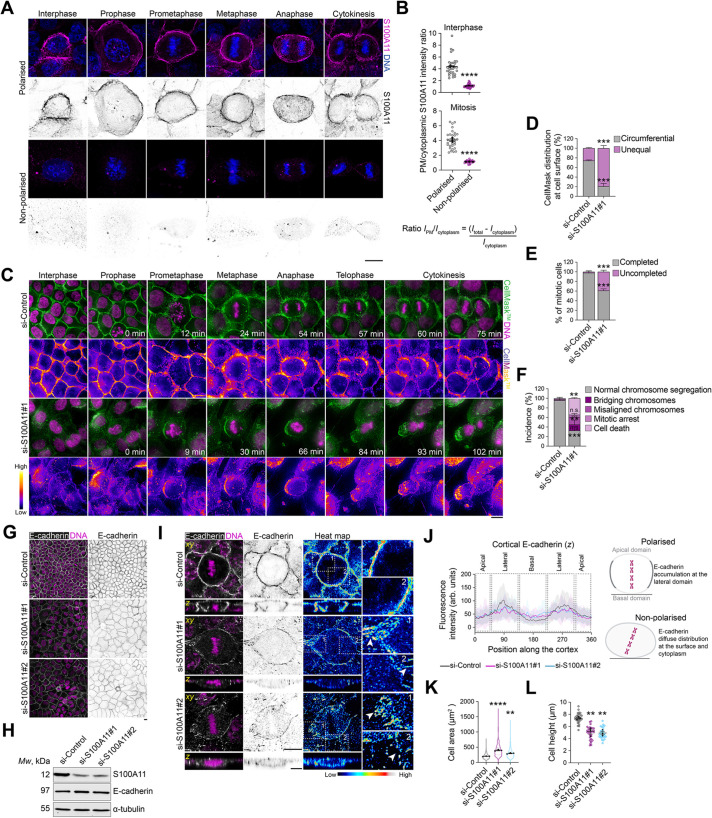
**S100A11 is required for correct plasma membrane remodelling and cell–cell adhesion integrity during mitosis.** (A) Confocal images of representative polarised and non-polarised MCF-10A cells stained for S100A11 (magenta) and counterstained with Hoechst 33342 (DNA, blue). Scale bar: 10 µm. (B) Plasma membrane (PM)-to-cytoplasmic S100A11 intensity ratio in interphase and mitotic cells (polarised, *n*=30 cells; non-polarised, *n=*30 cells). Unpaired two-tailed Student's *t*-test: interphase, ****P<0.0001; mitosis, ****P<0.0001. (C) Time-lapse images of representative MCF-10A cells transfected with control siRNA (si-Control) or siRNA targeting S100A11 (si-S100A11#1), labelled with CellMask (plasma membrane, green) and Hoechst 33342 (DNA, magenta) 10 min and 30 min before acquisition, respectively. Images showing CellMask intensity using a fire lookup table are presented beneath each time course. Scale bar: 10 µm. (D) Percentage of cells with the indicated CellMask labelling distributions at the cell surface during metaphase in siRNA-transfected cells (si-Control, *n*=28 cells; si-S100A11#1, *n=*59 cells). Unpaired two-tailed Student's *t*-test: circumferential, ***P=0.0008; unequal, ***P=0.0008. (E) Incidence of completed and uncompleted mitosis in siRNA-transfected cells (si-Control, *n*=28 cells; si-S100A11#1, *n*=59 cells). Unpaired two-tailed Student's *t*-test, ***P=0.0007. (F) Percentage of cells with the indicated mitotic defects in siRNA-transfected cells (si-Control, *n*=28 cells; si-S100A11#1, *n*=59 cells). Unpaired two-tailed Student's *t*-test: normal chromosome segregation, ***P=0.0002; misaligned chromosomes, **P=0.0012; bridging chromosomes, *P*=0.308; mitotic arrest, *P*=0.172; cell death, **P=0.002. (G) Confocal images of representative MCF-10A cells transfected with si-Control or siRNA targeting S100A11 (si-S100A11#1, si-S100A11#2), stained for E-cadherin (grey) and counterstained with Hoechst 33342 (DNA, magenta). Scale bar: 10 µm. (H) Western blotting of extracts from the indicated siRNA-transfected cells. Blots are stained for S100A11 and E-cadherin, and α-tubulin is shown as a loading control. Blots are representative of three experiments. (I) Confocal images of representative si-Control-, si-S100A11#1- or si-S100A11#2-transfected metaphase cells stained for E-cadherin (grey) and counterstained with Hoechst 33342 (DNA, magenta). Heat map images of E-cadherin signal are shown on the right, with dashed boxes marking regions shown in magnified views. Arrowheads indicate disassembled adherens junctions. Scale bars: 10 µm. (J) Mean cortical fluorescence intensity profiles of E-cadherin in siRNA-transfected metaphase cells (si-Control, n=30; si-S100A11#1, *n*=30; si-S100A11#2, *n*=27). Position is shown as angle (°). (K) Cell area in siRNA-transfected cells (si-Control, *n*=1699 cells; si-S100A11#1, *n*=634 cells; si-S100A11#2, *n*=1110 cells). Violin plots showing the distribution of data with mean (line), s.e.m. (bars) and quartiles (dashed lines) indicated. One-way ANOVA with Dunnett's test: ****P<0.0001; **P=0.002. (L) Cell height in siRNA-transfected cells (si-Control, *n*=30 cells; si-S100A11#1, *n*=30 cells, si-S100A11#2: *n*=29 cells). One-way ANOVA with Dunnett's test: **P=0.0032; **P=0.0019. All data are presented as mean±s.e.m. from three or four independent experiments. In B and L, coloured data points represent cells, black data points represent independent experiments. n.s., not significant. Source data are provided in Table S1.

**Fig. 5.**
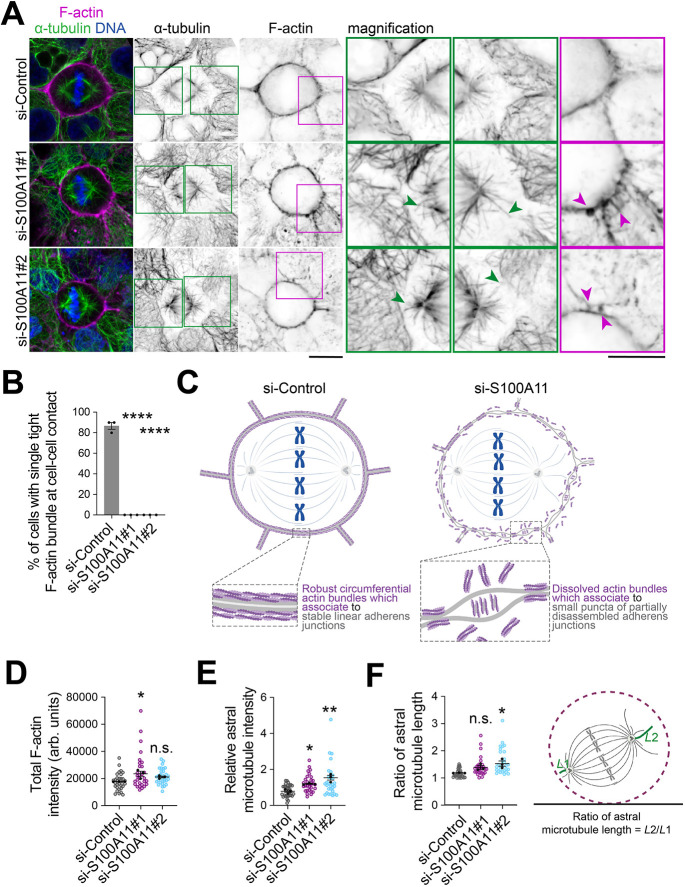
**S100A11 is required for F-actin and astral microtubule organisation during mitosis.** (A) Confocal images of representative MCF-10A cells treated with control siRNA (si-Control) or siRNA targeting S100A11 (si-S100A11#1, si-S100A11#2). Metaphase cells were stained for α-tubulin (green) and F-actin (magenta), and counterstained with Hoechst 33342 (DNA, blue). Boxes indicate regions shown in magnified views. Arrowheads indicate astral microtubules with aberrant length and shape (green) and dissolved actin bundles (magenta). Scale bars: 10 µm. (B) Percentage of cells with single tight F-actin bundle formation at cell–cell contacts in siRNA-transfected cells (si-Control, *n*=30 cells; si-S100A11#1, *n*=36 cells; si-S100A11#2, *n*=38 cells). One-way ANOVA with Dunnett's test: ****P<0.0001; ****P<0.0001. (C) Illustration showing F-actin bundles at cell–cell contacts and the effect of S100A11 knockdown on their assembly. (D) Fluorescence intensity of total F-actin in siRNA-transfected cells (si-Control, *n*=32 cells; si-S100A11#1, *n*=29 cells; si-S100A11#2, *n*=31 cells). One-way ANOVA with Dunnett's test: *P=0.019; n.s., *P*=0.185. (E) Relative fluorescence intensity of astral microtubules in siRNA-transfected cells (si-Control, *n*=31 cells; si-S100A11#1, *n*=30 cells; si-S100A11#2, *n*=30 cells). One-way ANOVA with Dunnett's test: *P=0.0251; **P=0.0015. (F) Ratio of astral microtubule length in siRNA-transfected cells (si-Control, *n*=29 cells; si-S100A11#1, *n*=29 cells; si-S100A11#2, *n*=29 cells). One-way ANOVA with Dunnett's test, n.s., *P*=0.130; *P=0.021. All data are presented as mean±s.e.m. from three independent experiments. In D–F, coloured data points represent mitotic cells, black data points represent independent experiments. Arb. units, arbitrary units; n.s., not significant. Source data are provided in Table S1.

We recently characterised the LGN cortical interactome in mitotic mammary epithelial cells, where we identified ANXA1 as a polarity cue that controls planar mitotic spindle orientation (Fankhaenel et al., 2023). Our proteomic screening also identified S100A11, which is an established partner of ANXA1 (Rintala-Dempsey et al., 2008). The function of S100A11 in mitosis has not been characterised previously. Our recent findings along with previous studies showing that S100A11 is required for plasma membrane remodelling by mediating a dynamic interplay between the plasma membrane and F-actin (Jaiswal et al., 2014; Mohammed et al., 2023 preprint), and the fact S100A11 interacts with E-cadherin (Guo et al., 2014), prompted us to test whether S100A11 function in the regulation of plasma membrane remodelling may influence cell–cell adhesion integrity and F-actin reorganisation during mitosis. Using immunofluorescence and confocal imaging, we conducted a first characterisation of the localisation of S100A11 during the cell cycle, revealing that S100A11 distributes in the cytoplasm and accumulates at the plasma membrane of mitotic polarised MCF-10A cells (Fig. 4A). Plasma membrane localisation of S100A11 was impaired in non-polarised cells (Fig. 4A,B). To examine the role of S100A11 in plasma membrane remodelling during mitosis, we performed live imaging in control and S100A11-depleted MCF-10A cells grown at optimal density, which we labelled with CellMask (Fig. 4C; Movies 10 and 11). We observed unequal distribution of CellMask in a vast majority of S100A11-depleted cells (si-S100A11#1, ∼79%; si-Control, ∼26%), which displayed a mesenchymal behaviour and asymmetric plasma membrane elongation during mitosis (Fig. 4C,D), similar to our observations of cells grown at low density. The proportion of cells that completed mitosis decreased significantly upon S100A11 knockdown (si-S100A11#1, ∼61%; si-Control, ∼98%; Fig. 4E). S100A11-depleted cells that completed mitosis displayed chromosome alignment defects and segregation defects in ∼16% and ∼67% of cells, respectively (Fig. 4F). Immunofluorescence and confocal imaging revealed that S100A11 knockdown impaired the localisation of E-cadherin at adherens junctions at 48 h after knockdown of S100A11, whereas the total cellular levels of E-cadherin did not change (Fig. 4G,H). These defects in cell–cell adhesion persisted at 72 h after depletion of S100A11 (Fig. S3). A close examination of the localisation of E-cadherin in metaphase cells showed that the protein accumulated in the cytoplasm and failed to cluster properly at adherens junctions of S100A11-depleted cells (Fig. 4I,J). This impaired cell–cell adhesion integrity and resulted in significant defects in the cytoarchitecture of S100A11-depleted cells (Fig. 4K,L). Consistent with this, S100A11 knockdown abrogated the assembly of single tight F-actin bundles at cell–cell contacts (Fig. 5A–C). Actin bundles ensure structural support of the plasma membrane (Zsolnay et al., 2020) and function as mechanosensors maintaining epithelial cell mechanics and integrity (Narayanan et al., 2015; Rückerl et al., 2017). S100A11 knockdown also impaired the organisation of cortical F-actin and its interaction with astral microtubules during metaphase, of which the length was also affected (Fig. 5D–F). Our results indicate that S100A11 is a key membrane-associated protein that coordinates plasma membrane remodelling with cell–cell adhesion maintenance and cell cortex organisation during mitosis.

### S100A11 forms a complex with E-cadherin and LGN to control mitotic spindle orientation

**Fig. 6.**
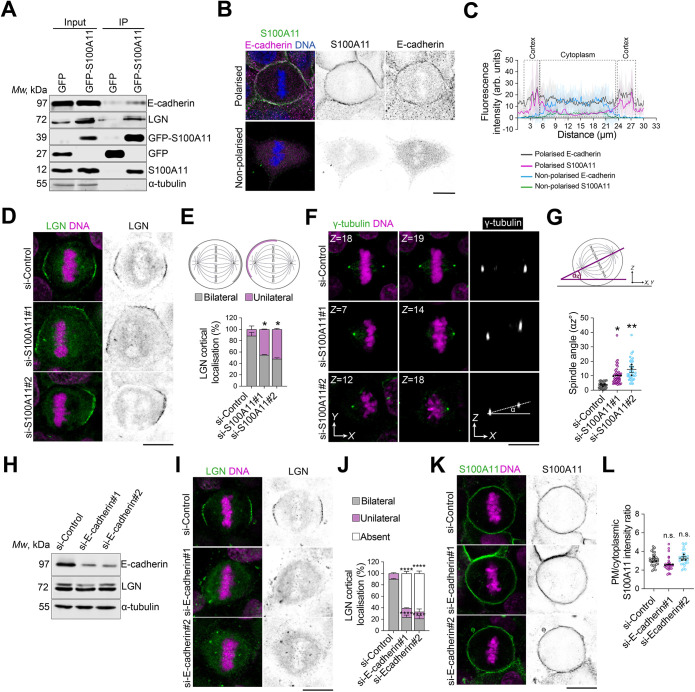
**S100A11 complexes with E-cadherin and LGN to regulate E-cadherin–LGN-mediated mitotic spindle orientation.** (A) GFP–S100A11, E-cadherin and LGN co-immunoprecipitate from clonal MCF-10A cells stably expressing GFP–S100A11, arrested in metaphase. Lysates of GFP–S100A11- or GFP-expressing cells were subjected to affinity purification with GFP-Trap beads. The immunoprecipitates (IP) were analysed by western blotting for the indicated proteins. Input lanes represent 10% of the total lysate. Blots are representative of three experiments. (B) Confocal images of representative polarised and non-polarised MCF-10A cells in metaphase stained for S100A11 (green) and E-cadherin (magenta), and counterstained with Hoechst 33342 (DNA, blue). Scale bar: 10 µm. (C) Mean cortical and cytoplasmic relative fluorescence intensity profiles of E-cadherin and S100A11 in ten representative polarised cells and ten representative non-polarised cells during metaphase. (D) Confocal images of representative MCF-10A cells transfected with control siRNA (si-Control) or siRNA targeting S100A11 (si-S100A11#1, si-S100A11#2). Metaphase cells were stained for LGN (green) and counterstained with Hoechst 33342 (DNA, magenta). Scale bar: 10 µm. (E) Percentage of cells with the indicated cortical localization patterns of LGN in siRNA-transfected cells (si-Control, *n*=64 cells; si-S100A11#1, *n*=64 cells; si-S100A11#2, *n*=49 cells). Two-way ANOVA with Tukey's test: *P=0.016; *P=0.0104. (F) Confocal images of representative si-Control-, si-S100A11#1- and si-S100A11#2-treated metaphase cells stained for γ-tubulin (green) and counterstained with Hoechst 33342 (DNA, magenta). *Z* section numbers are shown for *xy* views and spindle angle α*z* is indicated. Scale bar: 10 µm. (G) Mitotic spindle angle α*z* in siRNA-transfected metaphase cells (si-Control, *n*=37 cells; si-S100A11#1, *n*=41 cells; si-S100A11#2, *n*=36 cells). One-way ANOVA with Dunnett's test: *P=0.025; **P=0.002. (H) Western blotting of extracts from MCF-10A cells transfected with si-Control or with siRNA targeting E-cadherin (si-E-cadherin#1, si-E-cadherin#2) to detect E-cadherin and LGN, with α-tubulin shown as a loading control. Blots are representative of three experiments. (I) Confocal images of representative si-Control-, si-E-cadherin#1- and si-E-cadherin#2-treated metaphase cells stained for LGN (green) and counterstained with Hoechst 33342 (DNA, magenta). Scale bar: 10 µm. (J) Percentage of cells with the indicated cortical localisation of LGN in siRNA-transfected cells (si-Control, *n*=39 cells; si-E-cadherin#1, *n*=51 cells; si-E-cadherin#2, *n*=43 cells). Two-way ANOVA with Dunnett's test: bilateral, ****P<0.0001 and ****P<0.0001; unilateral: *P*=0.379 and *P*=0.891; absent: ****P<0.0001 and ****P<0.0001 for si-E-cadherin#1 and si-E-cadherin#2, respectively. (K) Confocal images of representative si-Control-, si-E-cadherin#1- and si-E-cadherin#2-treated metaphase cells stained for S100A11 (green) and counterstained with Hoechst 33342 (DNA, magenta). Scale bar: 10 µm. (L) Plasma membrane (PM)-to-cytoplasmic ratio of S100A11 fluorescence intensities in siRNA-transfected cells (si-Control, *n*=30 cells; si-E-cadherin#1, *n*=30 cells; si-E-cadherin#2, *n*=30 cells). One-way ANOVA with Dunnett's test: *P*=0.114 and *P*=0.589 for si-E-cadherin#1 and si-E-cadherin#2, respectively. All data are presented as mean±s.e.m. from two, three or four independent experiments. In G and L, coloured data points represent mitotic cells, black data points represent independent experiments. Arb. units, arbitrary units; n.s., not significant. Source data are provided in Table S1.

Direct binding of LGN to E-cadherin and its afadin-mediated interaction with cortical F-actin regulate the localisation and function of NuMA–dynein–dynactin to generate cortical forces that orient the mitotic spindle in epithelial cells (Carminati et al., 2016; Gloerich et al., 2017). Our results described above and previous findings of other labs showing that S100A11 interacts with E-cadherin (Guo et al., 2014) and F-actin (Shankar et al., 2010) prompted us to test whether S100A11 regulates LGN-mediated mitotic spindle orientation. First, we performed affinity purification to isolate the S100A11 complex from stable MCF-10A cells expressing GFP–S100A11 that were arrested in metaphase (Fig. 6A). GFP–S100A11 was distributed in the cytoplasm and localised to the cell surface throughout the cell cycle (Fig. S4A; Movie 12), which is consistent with recent studies of HeLa and U2OS osteosarcoma cells (Mohammed et al., 2023 preprint). We validated synchronisation efficiency by assessing the accumulation of phosphorylated histone H3 (Fig. S4B). Our pull-down assays combined with western blotting analysis revealed that GFP–S100A11 co-precipitates LGN and E-cadherin (Fig. 6A). Previously, endogenous S100A11 has been co-purified in a reciprocal manner with GFP–LGN (Fankhaenel et al., 2023) and E-cadherin–BirA* (Guo et al., 2014). Furthermore, we found that endogenous S100A11 colocalised with E-cadherin at the cell surface in polarised epithelial cells, whereas the two proteins co-accumulated in the cytoplasm of non-polarised cells (Fig. 6B,C). Next, we performed immunofluorescence and confocal imaging to evaluate the extent to which S100A11 affects the localisation of LGN at the lateral cortex during metaphase. S100A11 knockdown impaired the bilateral cortical distribution of LGN observed in control cells, with a significant increase in the proportion of S100A11-depleted cells displaying a unilateral accumulation of LGN (si-S100A11#1, ∼45%; si-S100A11#2, ∼52%; si-Control, ∼11%; Fig. 6D,E). These results further suggest that S100A11 regulates mitotic spindle orientation. We tested this hypothesis directly using immunofluorescence and confocal imaging and found that S100A11 knockdown resulted in mitotic spindle misorientation, with the spindle angle α*z* increasing from ∼4° in controls to ∼10° and ∼14° in cells treated with si-S100A11#1 and si-S100A11#2, respectively (Fig. 6F,G). Interestingly, E-cadherin knockdown impaired the recruitment of LGN to the cell cortex (Fig. 6H–J) but moderately affected S100A11 localisation at the plasma membrane (Fig. 6K,L), suggesting that S100A11 might act upstream of E-cadherin to control the mitotic spindle orientation machinery.

## DISCUSSION

**Fig. 7.**
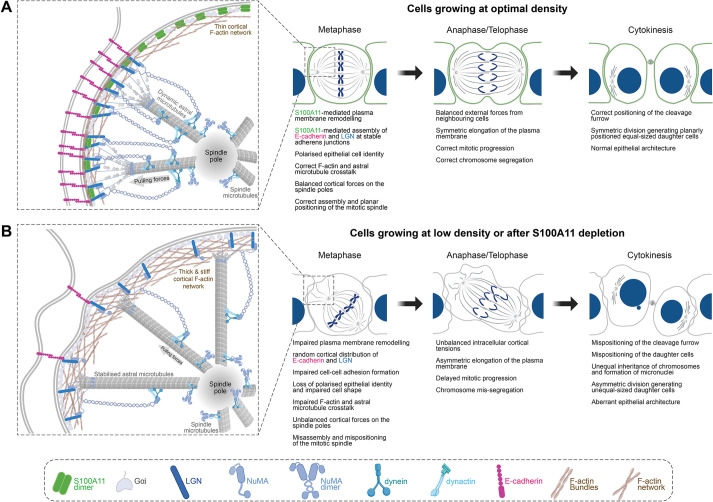
**Proposed model for the role of S100A11-mediated plasma membrane remodelling in the regulation of polarised epithelial cell divisions.** (A) S100A11 associates to the plasma membrane of polarised mammary epithelial cells grown at optimal density to regulate its remodelling during metaphase, which in turn controls mitotic cell shape. This, along with the formation of a complex between S100A11, E-cadherin and LGN, ensures correct organisation of the cortex and polarised accumulation of E-cadherin and LGN at the lateral cortex, thereby maintaining epithelial cell identity and ensuring proper LGN-mediated mitotic spindle assembly and orientation. Consequently, cells execute and achieve correct polarised divisions. (B) In cells grown at low density or at optimal density after S100A11 depletion, S100A11 is lost at the plasma membrane, which undergoes aberrant remodelling, impairing mitotic cell shape. This also impairs the localisation of E-cadherin and LGN at the cell cortex, which is thicker and stiffer. In these conditions cells lose their epithelial identity and fail to properly assemble and orient their mitotic spindle during metaphase. Subsequently, this results in aberrant mitosis mechanics, progression and outcome.

Polarised cell divisions are crucial for mammalian epithelial tissue differentiation, integrity and function by defining the fate and position of daughter cells (Lechler and Mapelli, 2021). Recent evidence has shown that epithelial polarity influences the orientation, progression and outcome of cell division, including how chromosomes segregate to daughter cells (Gloerich et al., 2017; Hart et al., 2017; Knouse et al., 2018; Lechler and Mapelli, 2021; Wang et al., 2018). However, most of our mechanistic knowledge of how cell shape dictates the dynamic progression of mammalian cell division has advanced largely from studies in non-polarised or isolated cells grown on adhesive micropatterns (Kiyomitsu and Cheeseman, 2013; Rizzelli et al., 2020; van Leen et al., 2020). While these studies have extensively described the influence of adhesive substrates and the extracellular matrix on the mechanics of mitosis by regulating the crosstalk between the cortical cytoskeleton and the mitotic machinery (Rizzelli et al., 2020; Taubenberger et al., 2020), how these mechanisms are coordinated with plasma membrane signalling and cell–cell adhesion remains poorly defined. Our present experiments in mammary epithelial cells uncover a functional interplay between plasma membrane remodelling and cell–cell adhesion that maintains epithelial cell identity for correct dynamics and outcome of polarised cell divisions. We further report that cell–cell adhesion assembly dictates plasma membrane remodelling and F-actin–astral microtubule crosstalk to control the shape and size of mitotic cells, which in turn ensures proper mitotic spindle dynamics and faithful chromosome segregation, as well as symmetric separation of daughter cells during cytokinesis. At the molecular level, we identify S100A11 as an upstream membrane-associated cue that coordinates correct plasma membrane remodelling with cell–cell adhesion maintenance and cortical cytoskeleton reorganisation during mitosis. We demonstrate that S100A11 complexes with E-cadherin and LGN to direct their polarised localisation to the lateral cortex for correct orientation, progression and outcome of mammary epithelial cell divisions (Fig. 7). Taken together, our findings shed new light on the function of the plasma membrane as a molecular signalling platform coordinating cell adhesion-mediated mechanochemical crosstalk between mitotic epithelial cells and their tissue environment to maintain their epithelial identity and execute error-free cell divisions.

Most studies addressing the role of external cues in the regulation of mammalian cell divisions have been carried out in isolated cells grown on adhesive micropatterns, a method pioneered by Bornens and Théry (Théry et al., 2007). These studies have shown that mitotic focal/substrate adhesion, maintained by integrin signalling, is key for correct chromosome and mitotic spindle dynamics and cytokinesis (Dix et al., 2018; Knouse et al., 2018; Lechler and Fuchs, 2005; Taneja et al., 2016). Nonetheless, cell–cell adhesions are essential for epithelial cells to acquire a defined shape and polarity, which are maintained to ensure correct execution of mitosis (Ragkousi and Gibson, 2014; Ramkumar and Baum, 2016; Taubenberger et al., 2020). Disruption of tissue architecture in several mammalian epithelial tissues, such as skin, liver, prostate and mammary gland, affects cell shape and polarity, leading to defects in chromosome segregation and mitotic dynamics (Knouse et al., 2018; Wang et al., 2018). Our experiments show that perturbation of cell–cell adhesion assembly in mammary epithelial cells cultured at low density abrogates E-cadherin localisation to the cell surface, which affects cell shape throughout mitosis. Our results suggest, but do not prove, that perturbation of cell–cell adhesion affects remodelling of the plasma membrane, which elongates asymmetrically to generate unequal-sized daughter cells at cytokinesis. This is in sharp contrast with studies in isolated HeLa cells, which have shown that anillin–RanGTP-dependent plasma membrane asymmetric elongation corrects mitotic spindle positioning defects during late anaphase and ensures symmetric cell division (Kiyomitsu and Cheeseman, 2013). Our observation that asymmetric elongation of the plasma membrane upon perturbation of cell–cell adhesion assembly results in mitotic spindle and sister chromatid mispositioning that persists until telophase indicates that additional molecular mechanisms coordinate plasma membrane remodelling and mitotic spindle dynamics in polarised epithelial cells. Remodelling of the plasma membrane in HeLa cells is accompanied by polar cortical blebbing, which recentres the mitotic spindle and releases cortical tensions to stabilise cell shape and ensure correct positioning of the cleavage furrow (Kiyomitsu and Cheeseman, 2013; Sedzinski et al., 2011). Our results in non-polarised mammary epithelial cells showing that blebbing does not rescue defects in positioning of the mitotic spindle, sister chromatids and the cleavage furrow reinforce a model whereby E-cadherin-mediated cell–cell adhesion is crucial for mitotic cells to sense and integrate external forces from neighbouring cells, which in turn decreases intracellular cortical tensions to ensure symmetric cell division. Thus, mammary epithelial cells must interact with their tissue environment to maintain their epithelial identity and native geometry to execute error-free mitosis. Importantly, one must be cautious when modelling and generalising some mechanisms of cell division; cellular identity (in this case, the epithelial origin) defines the response of mitotic cells to certain environmental cues, which might not be observed in non-epithelial cells.

We show that non-polarised mitotic mammary epithelial cells round up faster and are rounder as compared to polarised cells, further pointing to differences with findings in isolated cells (Serres et al., 2020) and the important role of cell–cell adhesion in the regulation of the mechanics of mitosis in epithelial cells. Reorganisation of the actomyosin cytoskeleton into a uniform, contractile cortical meshwork is a key mechanism for the generation of cortical tensions at the cell surface that drive mitotic rounding, which culminates during metaphase (Rizzelli et al., 2020; Taubenberger et al., 2020). Remarkably, we found that perturbation of cell–cell adhesion assembly leads to an increase in the thickness of cortical F-actin in metaphase cells, which correlates with efficient rounding. This contrasts with findings in isolated HeLa cells, where the thinning of cortical actin is crucial for generating cortical tensions that drive efficient mitotic rounding (Serres et al., 2020). We speculate that cortical actin thickening in non-polarised mitotic mammary epithelial cells acts as a compensatory mechanism that allows cells to overcome loss of tensions and traction forces that polarised cells receive through E-cadherin from neighbouring cells and the epithelial tissue layer. Consistent with this hypothesis, we show that polarised cells have a thinner F-actin cortex and adopt an elongated shape that is defined by neighbouring cells throughout mitosis, where the mitotic spindle aligns along the longest axis of the cell. E-cadherin interacts with actin to sense external mechanical forces and mediate their transduction into intracellular signalling (Buckley et al., 2014; Iskratsch et al., 2014; Lecuit and Yap, 2015; Yonemura et al., 2010). Loss of E-cadherin impairs F-actin integrity, contributing to morphological changes that induce epithelial-to-mesenchymal transition (EMT) (Chen et al., 2014), consistent with our observations showing that cells failing to assemble cell–cell adhesions acquire a mesenchymal behaviour. E-cadherin reduces cortical actomyosin contractility, ensuring correct mitotic spindle assembly and centrosome dynamics and maintaining epithelial integrity (Hidalgo-Carcedo et al., 2011; Rhys et al., 2018). Vinculin has been shown to connect E-cadherin to F-actin at adherens junctions to coordinate mitotic rounding with epithelial integrity maintenance (Monster et al., 2021). Interestingly, in our study, fast and efficient rounding up of non-polarised mammary epithelial cells did not prevent defects in mitotic spindle dynamics or mitosis progression and outcome. This is again different from findings in HeLa cells showing that efficient cell rounding ensures correct mitotic spindle assembly and chromosome segregation (Serres et al., 2020). This further points to the crosstalk between mitotic mammary epithelial cells and their neighbouring cells, through E-cadherin, to coordinate cell–cell adhesion with correct cortical actin reorganisation and mitotic mechanics. Consistent with this idea, rounding mitotic MDCK cells generate pushing forces against tissue confinement to create the space necessary for their division and proper mitotic spindle orientation and chromosome segregation (Sorce et al., 2015). Reciprocally, maintenance of cell–cell adhesion integrity allows neighbouring cells to exert tensions and traction forces that fine-tune mitotic cell rounding for correct mitosis mechanics and progression (Chanet et al., 2017; Lancaster et al., 2013; Sorce et al., 2015).

Correct crosstalk between actin and astral microtubules at the cell cortex is crucial for generating balanced cortical forces that ensure proper assembly and alignment of the mitotic spindle, as well as for coordinating chromosome segregation with cytokinesis to ensure error-free cell divisions (Dogterom and Koenderink, 2019; Lechler and Mapelli, 2021; van Leen et al., 2020). In addition to the effects on cortical F-actin rearrangement in non-polarised cells, our experiments reveal an increase in the length of astral microtubules, indicating their stabilisation. This results in mitotic spindle misorientation and excessive oscillations, which persist during anaphase. The increase in mitotic spindle length in non-polarised cells did not rescue spindle and chromatid centring defects, which is again different from findings in HeLa cells where larger mitotic spindles are centrally placed in cells undergoing asymmetric plasma membrane elongation to ensure the separation of equal-sized daughter cells at cytokinesis (Kiyomitsu and Cheeseman, 2013). Our results showing several defects in mitotic spindle assembly upon perturbation of cell–cell adhesion formation could explain the high incidence of chromosome mis-segregation and delayed mitotic progression. We also propose that our observed defects in mitotic spindle dynamics in non-polarised cells are likely due to the thinning of the subcortical actin cloud, which has been shown to associate with retraction fibres and to regulate the growth of astral microtubules and their interaction with cortical actin during mitosis (Inoue et al., 2019; Kwon et al., 2015; Yu et al., 2019). The vesicle-bound protein NDP52 (also known as CALCOCO2) has been shown to regulate the dynamics of the subcortical actin ring, which in turn acts on the growth of astral microtubules to regulate LGN-mediated mitotic spindle orientation and precise chromosome segregation (Yu et al., 2019). Similarly, the myosin 10 (also known as MYO10) motor protein associates with astral microtubules and works in parallel with dynein to regulate microtubule dynamic interaction with the cortex and orient centrosomes towards the subcortical actin cloud during mitosis (Kwon et al., 2015). During planar cell division, astral microtubule plus ends are coupled to E-cadherin junctions that are associated with the actin cortex (Dogterom and Koenderink, 2019). E-cadherin senses tensile forces from neighbouring cells at adherens junctions to pattern LGN at the cell cortex (Gloerich et al., 2017; Hart et al., 2017), while afadin concomitantly interacts with F-actin and LGN to regulate cortical F-actin reorganisation (Carminati et al., 2016), thereby promoting polarised cortical recruitment of dynein and anchoring of astral microtubules. Future studies will be key to characterise actin- and microtubule-associated proteins and crosslinkers that regulate the dynamics of F-actin and astral microtubules, and their crosstalk, in mitotic mammary epithelial cells and to determine how their function is coordinated with cell–cell adhesion.

Here, we propose a mechanism whereby the membrane-associated S100A11 protein acts as a molecular sensor of external cues that links plasma membrane remodelling to E-cadherin-dependent cell adhesion to coordinate cortex rearrangement and LGN-mediated mitotic spindle dynamics for correct progression and outcome of mammary epithelial cell divisions. Importantly, we show that S100A11 knockdown phenocopies the effect of perturbation of cell–cell adhesion formation in low-density cultures on plasma membrane remodelling and mitosis progression and outcome. Our observations showing S100A11 and E-cadherin colocalisation at the cell surface in polarised cells and their co-accumulation in the cytoplasm upon perturbation of cell–cell adhesion formation further point to a key role of S100A11 in coordinating the interplay between plasma membrane remodelling and E-cadherin function. Consistent with this, we reveal that S100A11 knockdown abrogates the polarised clustering of E-cadherin to the lateral cortex and impairs adherens junction formation, affecting the shape of mitotic and interphase mammary epithelial cells. Our observations that S100A11 depletion impairs cortical actin and astral microtubule organisation – which results in off-centred mitotic spindles in metaphase – also suggest that S100A11 function in the regulation of plasma membrane remodelling and cell–cell adhesion integrity is crucial for the previously described role of E-cadherin in directing localised reorganisation of F-actin and astral microtubules, as well as their crosstalk, at the sites of adherens junctions (Dogterom and Koenderink, 2019) to ensure correct assembly and positioning of the mitotic spindle. In the presence of Ca^2+^, S100A11 binds directly to actin to inhibit actin-activated myosin ATPase activity of smooth muscle cells, indicating a key role of S100A11 in the regulation of actin–myosin-mediated contractility (Zhao et al., 2000). S100A11 also associates with microtubules in keratinocytes, which facilitates translocation of S100A11 to the plasma membrane in a Ca^2+^-dependent manner (Eckert et al., 2004). Thus, we cannot rule out a direct effect of S100A11 on cortical actin and astral microtubules. Nonetheless, our immunoprecipitation experiments reveal that S100A11 forms a complex with E-cadherin and LGN in mitotic mammary epithelial cells, which corroborates recent proteomics studies showing that S100A11 co-purifies with LGN and E-cadherin in mammary epithelial cells and MDCK cells, respectively (Fankhaenel et al., 2023; Guo et al., 2014). These findings, along with those showing that F-actin complexes with LGN (Carminati et al., 2016), allow us to speculate that S100A11 might be part of the E-cadherin–F-actin–LGN complex to coordinate the crosstalk between adherens junctions, the cell cortex and the mitotic machinery. Interestingly, our observations showing loss of the polarised localisation of LGN to the lateral cortex in S100A11-depleted cells suggest that S100A11 is required for LGN cortical patterning rather than its cytoplasm-to-cortex recruitment, which is consistent with the function of its partner ANXA1 in the regulation of mitotic spindle planar orientation (Fankhaenel et al., 2023). It will be important to further dissect the mitotic functions of the S100A11–ANXA1 complex and its interaction with E-cadherin–F-actin–LGN. Related to this, we show that E-cadherin knockdown abrogates LGN recruitment to the cell cortex and impairs mitotic spindle orientation, as described previously (Gloerich et al., 2017; Wang et al., 2018), but does not affect the localisation of S100A11 to the plasma membrane, further reinforcing the idea that S100A11 acts upstream of E-cadherin to exert its function in mitosis. This also indicates that the cell–cell adhesion defect does not solely affect plasma membrane remodelling. Finally, our experiments do not reveal a localisation of S100A11 on the mitotic spindle or chromosomes, indicating that the effect of S100A11 depletion on chromosome segregation fidelity is a result of impaired cortical and plasma membrane remodelling, which is consistent with recent studies linking cortical organisation and cell geometry to faithful chromosome segregation (di Pietro et al., 2017; Knouse et al., 2018; Yu et al., 2019). Taken together, our findings identify S100A11 as a membrane-associated molecular landmark bridging plasma membrane remodelling, E-cadherin clustering to adherens junctions, cortical actin–astral microtubule dynamic crosstalk and the LGN mitotic spindle machinery for correct orientation, progression and outcome of mammary epithelial cell divisions. Further mechanistic studies will be key to understanding how S100A11 participates in the transduction of external cues into intracellular signalling and determining how this is spatiotemporally coordinated with plasma membrane remodelling and E-cadherin dynamics during mitosis.

In conclusion, our study in mammary epithelial cells reveals an important S100A11-dependent functional dynamic interplay between the plasma membrane and E-cadherin-mediated cell adhesion to control cortical cytoskeleton reorganisation and LGN-mediated mitotic machinery, thereby ensuring error-free cell divisions and correct shape and positioning of daughter cells. While the plasma membrane is established as a physical interface linking external cues to intracellular signalling during cell division (Rizzelli et al., 2020), our findings point to a key role of the plasma membrane as a molecular platform for components such as S100A11 that act as mechanical sensors controlling the mechanochemical crosstalk between extracellular and intracellular signalling for correct execution of polarised cell divisions. It will be important for future work to characterise the proteome and lipidome of the plasma membrane to identify factors that bridge plasma membrane remodelling and epithelial polarity, and to dissect how they coordinate these with the mitotic machinery. Remarkably, our experiments in non-transformed mammary epithelial cells devoid of oncogenic triggers demonstrate that perturbation of cell–cell adhesion is sufficient to induce mitotic and cytoarchitectural defects that are known as major contributors to developmental disorders and carcinogenesis (Lechler and Mapelli, 2021; Matthews et al., 2020; Taubenberger et al., 2020). Consistent with this, loss of E-cadherin in the normal prostate epithelium results in mitotic spindle and cell behaviour defects that lead to carcinogenesis (Wang et al., 2018). Chromosome instability and chromosome segregation defects have been linked to EMT (Comaills et al., 2016; Matthews et al., 2020; Roschke et al., 2008). Our findings in non-transformed mammary epithelial cells suggest that perturbation of cell–cell adhesion resulting in impaired epithelial identity represents a key initiating event leading to mitotic spindle dynamics and chromosome segregation defects that are known to drive epithelial malignant transformation (Ramkumar and Baum, 2016). Further studies using three-dimensional cultures and mouse models will be crucial for elucidating how polarised cell divisions are coordinated with external cues to drive mammary epithelial morphogenesis and carcinogenesis, and for understanding how this is defined by polarised epithelial cell identity.

## MATERIALS AND METHODS

### Cell lines

MCF-10A cells are spontaneously immortalised, non-transformed human mammary epithelial cells, obtained from ATCC (CRL-10317). Cells were cultured in Dulbecco's Modified Eagle Medium/F12 (DMEM/F12; Invitrogen), supplemented with 5% donor horse serum (Gibco), 20 ng/ml human EGF (Sigma, E9644), 0.5 μg/ml hydrocortisone (Sigma, H0888), 10 μg/ml insulin (Sigma, I1882), 100 ng/ml cholera toxin (Sigma, C8052), 50 U/ml penicillin and 50 μg/ml streptomycin (Life Technologies), and 500 ng/ml amphotericin B (Gibco, 11510496), at 37°C in a humidified 5% CO_2_ atmosphere. HEK293 cells (ATCC; CRL-3216) provided by Melissa Andrews (University of Southampton, UK) were cultured in DMEM with high glucose, sodium pyruvate and L-glutamine (Gibco), supplemented with 10% foetal bovine serum (FBS; Gibco) and 50 U/ml penicillin and 50 μg/ml streptomycin (Life Technologies), at 37°C in a humidified 5% CO_2_ atmosphere. Cell lines were periodically tested for mycoplasma contamination.

### siRNAs and transfections

Transient knockdown of S100A11 and E-cadherin in MCF-10A cells was performed by transfection of MISSION Predesigned (Sigma) or custom-made siRNAs, respectively. To knockdown S100A11, the following siRNAs were used: SASI_Hs01_00164495 (si-S100A11#1) and SASI_Hs01_00164498 (si-S100A11#2). To knockdown E-cadherin, the following siRNAs were used: si-E-cadherin#1 (sense, 5′-CAUCUUGACUAGGUAUUGUCU-3′; antisense, 5′-AGACAAUACCUAGUCAAGAUG-3′) and si-E-cadherin#2 (sense, 5′-GAGAGAGUUUCCCUACGUAUA-3′; anti-sense, 5′-UAUACGUAGGGAAACUCUCUC-3′). All experiments using siRNAs were carried out in the presence of siGENOME RISC-Free (Dharmacon), used as a negative control for siRNA experiments (si-Control). MCF-10A cells were transfected with a final concentration of 50 nM of siRNAs diluted in Opti-MEM Reduced Serum Medium (Gibco) using Lipofectamine RNAiMAX (Invitrogen), following the manufacturer's protocol. Transfected cells were incubated for 48 h or 72 h before they were live imaged, lysed, or fixed and immunoprocessed.

### Retroviral constructs and generation of stable cell lines

Retroviral constructs were used to transduce MCF-10A cells. Lifeact–mCherry was obtained from Addgene (pTK93_Lifeact-mCherry; Addgene, 46357). pTK14-GFP-S100A11 was cloned as follows. First, the human S100A11 sequence was obtained from the NCBI database (reference sequence: NM005620.2), and the S100A11 cDNA flanked by XhoI and SacII restriction digestion site sequences was synthesised in a pEX-A128 plasmid (Eurofins Scientific). Second, the synthesised S100A11 was removed from the pEX-A128 plasmid by restriction digestion with XhoI and SacII (New England Biolabs), then cloned into a pTK14-GFP plasmid (Fankhaenel et al., 2023). Correct insertion was verified by Sanger sequencing.

Generation of stable MCF-10A cell lines was performed using retroviral transduction. Retroviruses were prepared in HEK293 cells by calcium phosphate co-transfection of 4×10^6^ cells in 8 ml of culture medium with 10 μg retroviral plasmid, 5 μg packaging plasmid pUMVC (Addgene, 8449), 6.5 μg envelope plasmid pCMV-VSV-G (Addgene, 8454). Virus particles were collected 48 h after transfection then filtered through a 0.45 μm syringe filter and used to infect MCF-10A cells in the presence of 8 μg/ml polybrene (Sigma). Clones of interest were selected using 1 μg/ml puromycin (Sigma). All plasmids and cell lines are available upon request.

### Cell cycle synchronisation

MCF-10A cells were treated with 9 μM RO-3306 (Sigma, SML0569), dissolved in DMSO, for 18 h to allow CDK1 inhibition and synchronise cells in G2/M phase. To further arrest cells in metaphase, MCF-10A cells were released from the G2/M block by washing three times in pre-warmed drug-free medium and incubated in fresh medium for 35 min.

### Cell extracts and immunoblotting

MCF-10A cells were lysed in NP-40 lysis buffer [50 mM Trizma hydrochloride (Tris-HCl), pH 7.4; 250 mM NaCl; 5 mM ethylenediaminetetraacetic acid (EDTA); 50 mM NaF; 1 mM Na_3_VO_4_; 1% Nonidet P-40 (NP40)], supplemented with protease inhibitor cocktail (Sigma, P2714). Protein concentration of lysates was determined using a Pierce BCA protein assay (Thermo Fisher Scientific). Proteins were subjected to sodium dodecyl sulphate polyacrylamide gel electrophoresis (SDS-PAGE) and subsequent western blot analysis. The following primary antibodies were used: anti-α-tubulin DM1A (0.2 µg/ml; Sigma, T6199), anti-E-cadherin (1:500; Abcam, ab76055), anti-S100A11 (1:500; Proteintech, 10237-1-AP), anti-LGN (1:500; Sigma, ABT174), anti-phospho-histone H3 (0.2 µg/ml; Sigma, 06-570) and anti-GFP (2 μg/ml; Invitrogen, A-11122). Secondary antibodies conjugated to horseradish peroxidase (Invitrogen, 32430 and 32460) were used at 1:10,000. Proteins were visualised using SuperSignal West Pico PLUS Chemiluminescent Substrate (ECL) (Thermo Fisher Scientific), followed by imaging using a Syngene PXi detection system Scanner (Syngene). Images of uncropped blots are shown in Fig. S5.

### Immunoprecipitation

Clonal MCF-10A cells stably expressing pTK14-GFP-S100A11 or pTK14-GFP were plated in 15 cm dishes and washed twice with ice-cold PBS before protein extraction, for which ∼10×10^7^cells were lysed in a mild lysis buffer (50 mM Tris-HCl, pH 7.4, 150 mM NaCl, 0.5 mM EDTA, 10 mM NaF, 1 mM Na_3_VO_4_, 0.5% NP40) containing protease inhibitor cocktail (Sigma, P2714). Cell lysates were cleared by centrifugation at 17,000 ***g*** for 30 min at 4°C. Co-immunoprecipitation was performed using a GFP-Trap Kit (Chromotek, gtma-20) following the manufacturer's instructions. For immunoblotting, washed beads were eluted by boiling in Laemmli sample buffer (Bio-Rad) containing 5% 2-mercaptoethanol.

### Immunofluorescence

The following primary antibodies were used: anti-E-cadherin (1:200; Fisher, 13-1900), anti-S100A11 (10 µg/ml; Proteintech, 10237-1-AP), anti-LGN (1:200; Sigma, ABT174), anti-α-tubulin DM1A (1 µg/ml; Sigma, T6199), anti-γ-tubulin AK-15 (1:300; Sigma, T3320). Alexa Fluor 555-conjugated phalloidin (1:50, Life Technologies; A34055) was used to label F-actin. Secondary antibodies (Life Technologies) used were goat anti-mouse (A-32723 and A-21125), anti-rabbit (A-11037 and A-11008) and anti-rat (A-11007) conjugated to Alexa Fluor 488, Alexa Fluor 594 or Alexa Fluor 647, at 5 µg/ml.

To visualise α-tubulin and γ-tubulin, MCF-10A cells were fixed in ice-cold anhydrous methanol (Sigma) for 5 min followed by fixation with 4% paraformaldehyde (PFA; Fisher) for 10 min. PFA was prepared in PHEM [60 mM piperazine-1,4-bis(2-ethanesulfonic acid) (PIPES), 25 mM 4-(2-hydroxyethyl)-1-piperazineethanesulfonic acid (HEPES), 10 mM ethylene glycol-bis(β-aminoethyl ether)-N,N,N′,N′-tetraacetic acid (EGTA) and 4 mM MgSO_4_] diluted in PBS. Cells were washed with PBS, then permeabilised with 0.1% Triton X-100–PBS (Sigma) for 10 min. Cells were blocked with 10% goat serum (Sigma) and 1% bovine serum albumin (BSA; Sigma) in 0.1% Triton X-100–PBS for 1 h. To visualise E-cadherin, S100A11 and LGN, cells were fixed in ice-cold anhydrous methanol for 10 min. Cells were then washed with PBS and incubated in 10% goat serum and 1% BSA in 0.1% Triton X-100–PBS for 1 h. To visualise cortical F-actin and astral microtubules, cells were fixed 10 min in 3% PFA and 0.25% glutaraldehyde (Sigma) in 0.2% NP40 (Sigma) diluted in Brinkley Buffer 1980 (BRB80; 80 mM PIPES, 1 mM MgCl_2_ hexahydrate and 1 mM EGTA). Cells were blocked in 0.1% NH_4_Cl (Sigma) in BRB80 for 10 min, followed by two washes in BRB80 for 5 min each. Cells were then blocked in 3% BSA in 0.2% NP40–BRB80. For all immunostaining, cells were incubated with primary antibodies at 4°C overnight. Cells were washed and incubated with appropriate secondary antibodies for 1 h at room temperature. Finally, cells were counterstained with 25 µg/ml Hoechst 33342 (Sigma) and mounted with Vectashield antifade mounting medium (Vector Laboratories).

### Quantitative confocal microscopy

Immunofluorescence images were captured with an inverted Leica TCS SP8 inverted laser scanning microscope (Leica Microsystems) using a 63× glycerol immersion objective (63× HC Plan/Apo CS2 1.30 NA). *Z*-stacks at 16-bit depth and 2048×2048 pixels were collected at 0.2 or 0.3 μm intervals. Images were processed with Fiji software (Schindelin et al., 2012). Astral microtubule images were denoised using the MATLAB-based ND-Safir software (Boulanger et al., 2010).

Cortical fluorescence intensity of E-cadherin in metaphase cells was measured in Fiji using a custom macro as described previously (di Pietro et al., 2017). The macro measured the pixel value at the cell cortex, providing 180 measurements with 2° intervals around the defined cell cortex. Fluorescence intensity values were reported along the cortex starting (and finishing) at a point facing the metaphase plate. For measurements along the *xy* axis, positions 0° and 180° face the chromosome plate (central cortex), and position 90° and 270° face the spindle poles (lateral cortex): 180 positions were scanned (every 2°) along the cortex. Along the *z* axis, positions 0° and 360° indicate the apical cortex, 90° and 270° indicate the lateral cortex, and 180° indicates the basal cortex. To account for more elongated cell shapes, a line was drawn along the cell contour using the Freehand tool, and the macro fits an ellipse to this contour. Fluorescence intensities were calculated along a 30-pixel radial line overlapping the cortex, and the maximum intensity was reported. Measurements were taken at 180 successive positions (every 2°), starting from (and finishing) at the short axis of the ellipse. Background values were subtracted from all measured fluorescence intensities.

To measure relative fluorescence intensities of E-cadherin and S100A11 at the cell cortex and cytoplasm, a 30-pixel line was drawn across the lateral surface and the cytoplasm using Fiji software. The line scan function of Fiji was used to reveal the relative fluorescence intensity across the line.

Measurement of F-actin fluorescence intensities was performed on 8-bit images, generating a selection for measuring total F-actin fluorescence intensity (*I*_total actin_). Cortical F-actin fluorescence intensity (*I*_cortical actin_) was measured similarly, with the use of the freehand tool to draw around the cortex of the cell. Subcortical F-actin fluorescence intensity (*I*_subcortical actin_) was determined by subtracting cortical F-actin fluorescence from total F-actin fluorescence. Background values were subtracted for correct F-actin fluorescence intensity measurement on maximum projection images. Actin cloud penetration length was measured by drawing a line between the subcortical and spindle pole areas at both sides of the cell poles, and then an average value was calculated. Quantification of F-actin organisation at adherens junctions was performed by analysing the clustering of F-actin at sites of cell–cell contacts, which was defined by the formation of F-actin bundles.

Like F-actin, total, plasma membrane and cytoplasmic S100A11 fluorescence intensities were measured. The ratio of plasma membrane-to-cytoplasmic S100A11 fluorescence intensities was measured using Fiji software. Maximum projections of images were generated, and the fluorescence intensity of the whole cell (*I*_total_) and the cytoplasm (*I*_cytoplasm_) were measured. Background signal was subtracted, and the plasma membrane-to-cytoplasm fluorescence intensity ratio of S100A11 (*I*_PM_/*I*_cytoplasm_) was calculated as *I*_PM_/*I*_cytoplasm_=(*I*_total_−*I*_cytoplasm_)/*I*_cytoplasm_.

Astral microtubule intensity (α-tubulin signal) was measured using Fiji software. Maximum projections of images were generated, and the fluorescence intensity of the whole cell (*I*_total_) and the spindle (*I*_spindle_) excluding spindle poles with astral microtubules were measured. Background signal was subtracted, and the relative fluorescence intensity of astral microtubules (*I*_astral, rel_) was calculated as *I*_astral, rel_=(*I*_total_−*I*_spindle_)/*I*_spindle_. Length of astral microtubules was measured by drawing a line along the astral microtubule extending towards the cell cortex on both sides of the poles. Similarly, the pole-to-cortex distance was measured by drawing a line towards the closest cell cortex in line with the spindle axis.

Cell segmentation and quantification of cell morphology were performed in cells stained for E-cadherin using the Fiji MorphoLibJ plugin, as described previously (Legland et al., 2016). Cell height was quantified in Fiji by drawing a straight line between the apical and basal membranes of cells viewed along the *z* axis.

Mitotic spindle orientation was measured using Fiji in metaphase cells stained for γ-tubulin. The spindle axis was defined by drawing a 30-pixel line across both spindle poles and repositioned along the *z* axis. The spindle axis angle α*z* was measured in respect to the substratum using the angle tool.

### Quantitative live-cell imaging

MCF-10A cells, or GFP–S100A11- or Lifeact–mCherry-expressing cell lines were plated in 27 mm glass-bottomed dishes (Nunc). Prior to imaging, cells were incubated in cell culture medium supplemented with 100 ng/ml Hoechst 33342 (Sigma) for 30 min to visualise DNA. When the plasma membrane or mitotic spindles were observed, cells were further incubated in cell culture medium supplemented with CellMask Deep Red Plasma Membrane Stain (Thermo Fisher Scientific # C10046) at 1:1000 dilution for 10 min or with 100 nM SiR-tubulin (Spirochrome, 251SC002) for 3 h. Cells were imaged at 37°C in CO_2_ independent medium (Gibco) using a DeltaVision Elite microscope (GE Healthcare) coupled to a sCMOS max chip area 2048×2048 camera (GE Healthcare). For each recording, image stacks at 0.6 μm increments in 1024×1024 format were acquired using a PlanApo 60×/1.42 Oil immersion objective (Olympus) with 2×2 binning. Images were taken at three stage positions every 3 min for 3 or 5 h. Exposure times were 200 msec and 5% laser power for GFP, 50 msec and 2% laser power for mCherry, 80 msec and 2% laser power for labelled DNA and microtubules, and 50 msec and 2% laser power for labelled plasma membrane using the DAPI-FITC-mCh-Cy5 filter set. Images were deconvolved using the DeltaVision software SoftWoRx and further processed using Fiji.

Plasma membrane dynamic remodelling was visualised by following the distribution of CellMask labelling at the cell surface in successive timeframes, where it displayed circumferential, unilateral or bilateral accumulation in mitotic cells. Plasma membrane elongation was quantified by merging images of anaphase and telophase using Fiji software. Then, elongation of the plasma membrane at the cell cortex of the newly formed daughter cells was assessed. Equal membrane elongation of the newly generated cells is considered symmetric elongation, whereas unequal elongation is considered asymmetric elongation. Measurement of the area of daughter cells, expressed in µm^2^, was performed using the freehand tool in Fiji to highlight the cortex of daughter cells at cytokinesis. The ratio of daughter cell area was calculated by dividing the largest cell area by the smallest cell area. Chromosome-to-cell cortex distance was measured by drawing a line from the middle of the sister chromatid cluster to the polar cell cortex during telophase using the line tool in Fiji. The ratio of chromosome-to-cell cortex distance was calculated by dividing the longest distance by the shortest distance for each cell at telophase. The number of blebs during anaphase-to-telophase transition was determined using the 3D objects counter tool in Fiji.

Oscillations of the mitotic spindle were calculated from time-lapse videos of cells labelled with SiR-tubulin and Hoechst 33342. Metaphase spindle angles in the *z* (α*z*) and *xy* (α*xy*) planes were manually determined for every frame using Fiji software. Measurements of the spindle angle axis in the *z* plane were performed as described above for fixed cells. Spindle angles were reported as positive values unless spindle poles changed direction. In this case, angles were displayed as negative values. Mitotic spindle orientation in the *xy* plane was measured by drawing a line crossing both spindle poles of the first frame of metaphase. This line was embedded in all the following frames to mark the initial position of the mitotic spindle. In the next frame, another line was overlaid across the poles to define the new spindle axis. The angle between the initial spindle position and the current spindle axis was measured using the angle tool. Spindle movement directions were considered by reporting spindle rotations clockwise as positive angles and reverse movements as negative angles. Spindle angle deviations greater than 10° between two frames were counted as oscillations. The oscillation index was determined as the percentage of oscillations in respect to the total number of frames.

Quantification of actin fluorescence from time-lapse videos of Lifeact–mCherry-expressing cells in prometaphase and metaphase was performed as described previously (Mahlandt and Goedhart, 2022). Ratios of prometaphase-to-metaphase total, cortical and subcortical actin were calculated. Cell morphology and roundness [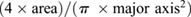] quantification was performed using the shape descriptors plugin in Fiji. Rounding timing was determined by analysing successive frames of the time-lapse videos. The full width at half maximum (FWHM) of cortex intensity profiles was used to assess the thickness of cortical actin. A straight line with the same length was drawn across the cortical actin in all data analysed (cortical actin was centred at the middle of the line). Then, a plot profile was generated using Fiji, and the FWHM calculated using Origin (Pro) Version 2021b (https://www.originlab.com/2021).

### Statistical analysis

All statistical analysis were performed with GraphPad Prism 9.1.2 software. Graphs were created using GraphPad Prism 9.1.2 software and RStudio (1.2.5033 version). Multiple groups were tested using analysis of variance (ANOVA) with post hoc Tukey or Dunnett tests, and comparisons between two groups were performed using *t*-tests. Data are shown as mean± s.e.m. from three or four independent experiments. *P*≤0.05 was considered statistically significant. Asterisks indicate levels of significance (*P≤0.05; **P≤0.01; ***P≤0.001; ****P≤0.0001). More specific details about the number of experiments and objects counted and statistical tests performed are indicated in the figure legends.

## Supplementary Material

10.1242/joces.261701_sup1 Supplementary information

Table S1.
