# Supplementary material for: Interplay between the plasma membrane and cell–cell adhesion maintains epithelial identity for correct polarised cell divisions
Source: J Cell Sci. 2023 Nov 28;137(5):jcs261701. doi: 10.1242/jcs.261701 (PMC10729819; doi:10.1242/jcs.261701)
Supplement: Supplementary information [file joces-137-261701-s1.pdf]

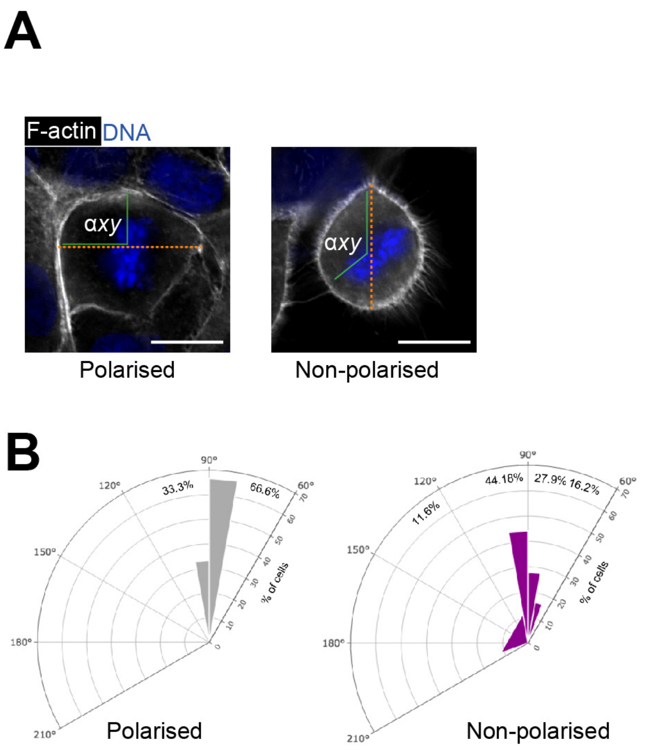

Fig. S1. The mitotic spindle aligns following Hertwig's rule in polarised cells but not in non-polarised cells. (A) Confocal images of representative polarised and non-polarised MCF-10A cells stained for F-actin (grey) and counterstained with Hoechst 33342 (DNA, blue). The orange dashed line indicates the long axis of the cell. The angle α*xy* indicates the orientation of the metaphase plate relative to the long axis of the cell. Scale bar, 10 μm. **(B)** α*xy* angle frequencies in polarised and non-polarised cells (polarised: n = 45 cells; non-polarised: n = 40 cells, 3 independent experiments).

Journal of Cell Science • Supplementary information

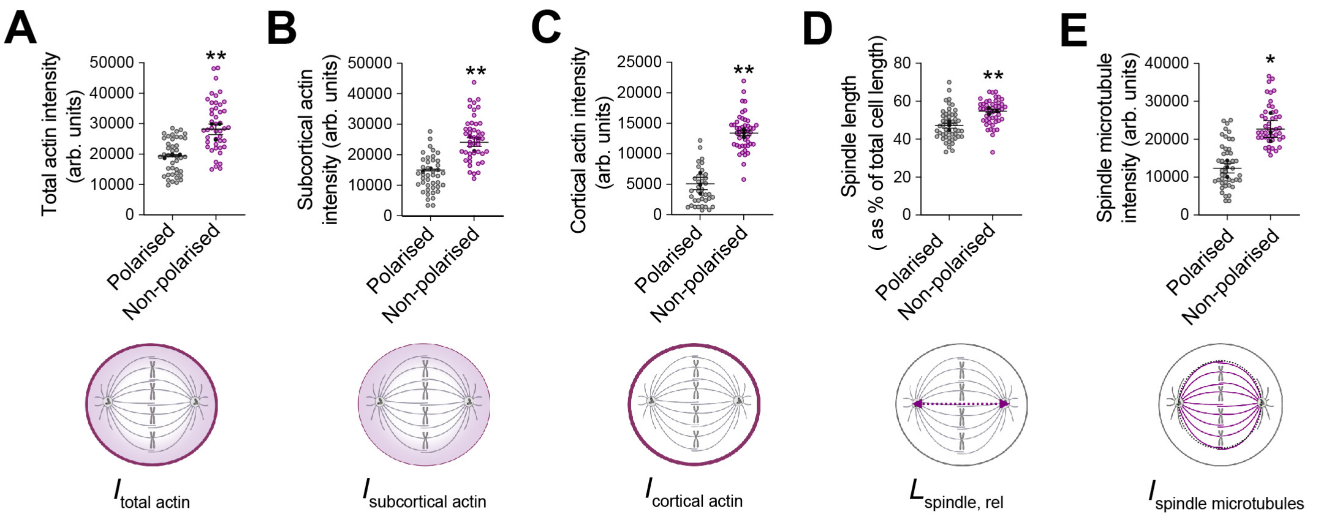

Fig. S2. Perturbation of cell-cell adhesion formation affects F-actin organisation and mitotic spindle assembly in fixed metaphase cells. (A-C) Fluorescence intensity of total F-actin, subcortical F-actin, and cortical F-actin in polarised and non-polarised MCF-10A cells (polarised: n = 42 cells; non-polarised: n = 45 cells). Two-sided t-test, total: **P = 0.0082; subcortical: **P = 0.0035; cortical: **P = 0.0015. **(D)** Spindle length in metaphase (polarised: n = 51 cells; non-polarised: n = 47 cells). Two-sided t-test, **P < 0.0094. **(E)** Relative fluorescence intensity of spindle microtubules (polarised: n = 46 cells; non-polarised: n = 44 cells). Two-sided t-test, *P = 0.0147. All data are presented as mean ± s.e.m. from 3 or 4 independent experiments. Source data are provided as a Source Data file.

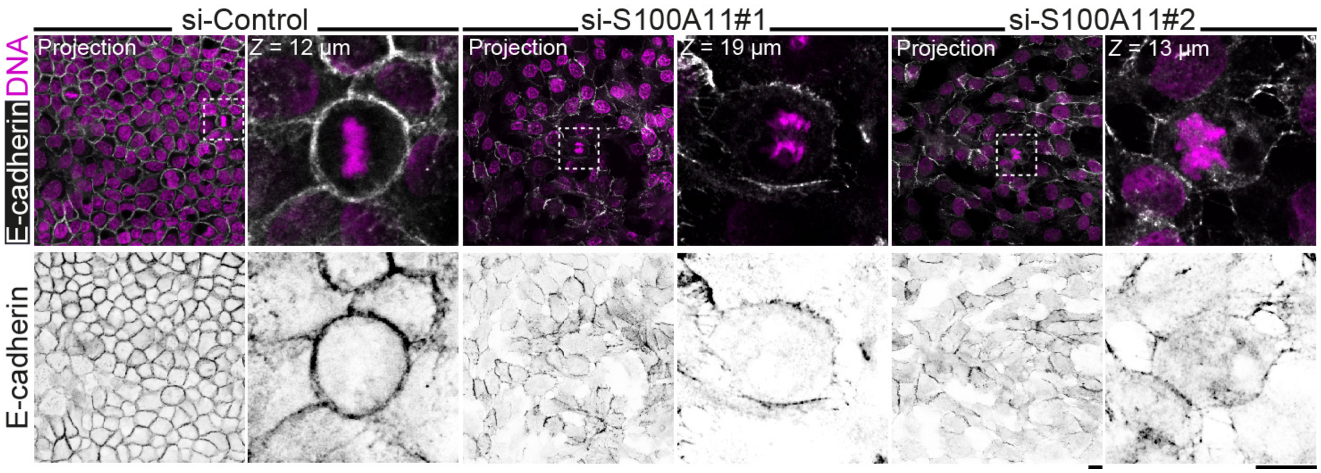

Fig. S3. Defects in cell-cell adhesion persist at 72 h post-S100A11 knockdown. Confocal images of representative si-Control-, si-S100A11#1, si-S100A11#2-treated cells stained for E-cadherin (grey) and counterstained with Hoechst 33342 (DNA, magenta). Scale bar, 10 µm.

Journal of Cell Science • Supplementary information

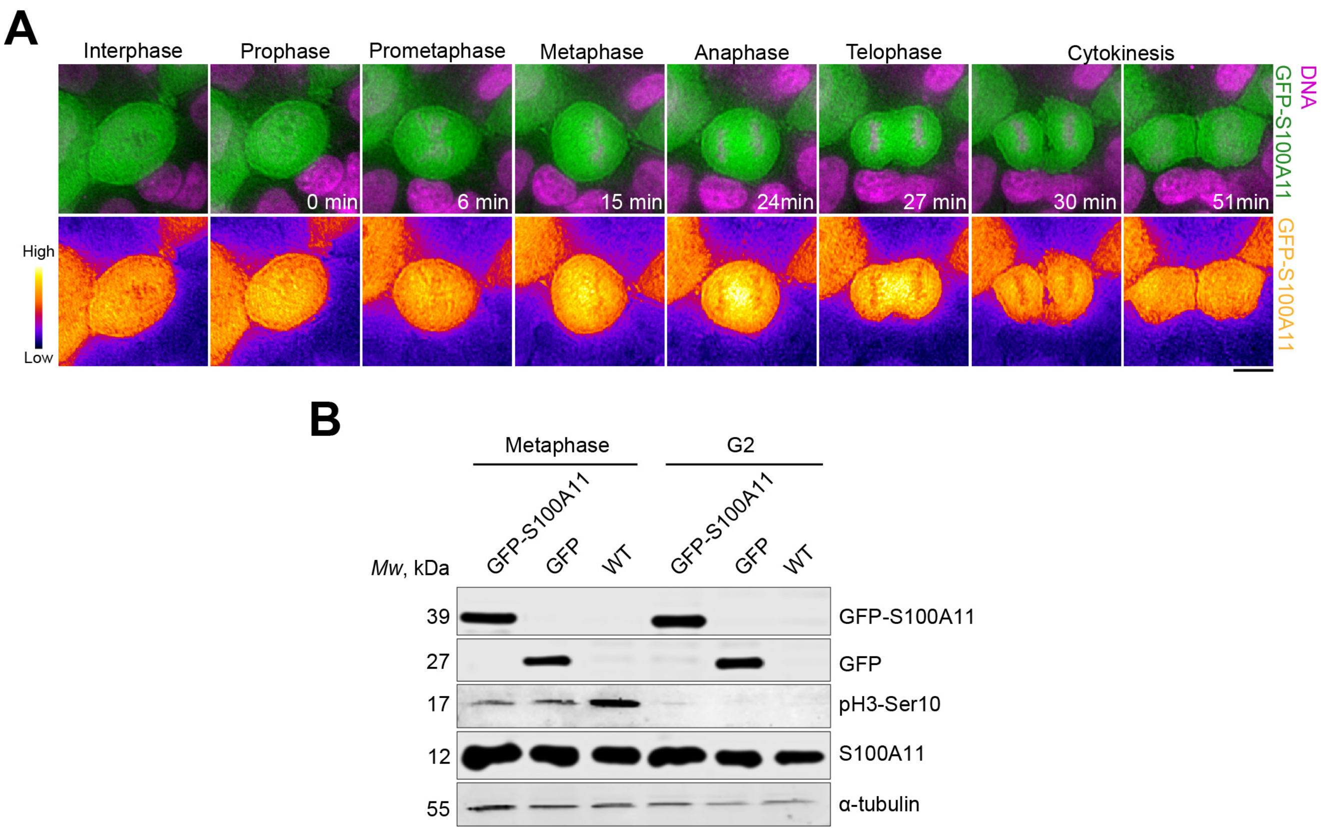

Fig. S4. Validation of the cell-cycle synchronisation protocol in mammary epithelial cells. (A) Time-lapse images of representative clonal MCF-10A cells stably expressing GFP-S100A11 (green). DNA is labelled with Hoechst 33342 (magenta). Scale bar, 10 µm. **(B)** Western blotting of extracts from wild type MCF-10A and clonal MCF-10A stably expressing GFP-S100A11 or GFP cells, synchronised in G2 or metaphase. Blots are stained for GFP, S100A11 and phospho-histone H3 (pH3-ser10), and α-tubulin as a loading control (3 independent experiments). Source data are provided as a Source Data file.

Journal of Cell Science • Supplementary information

Figure 4H

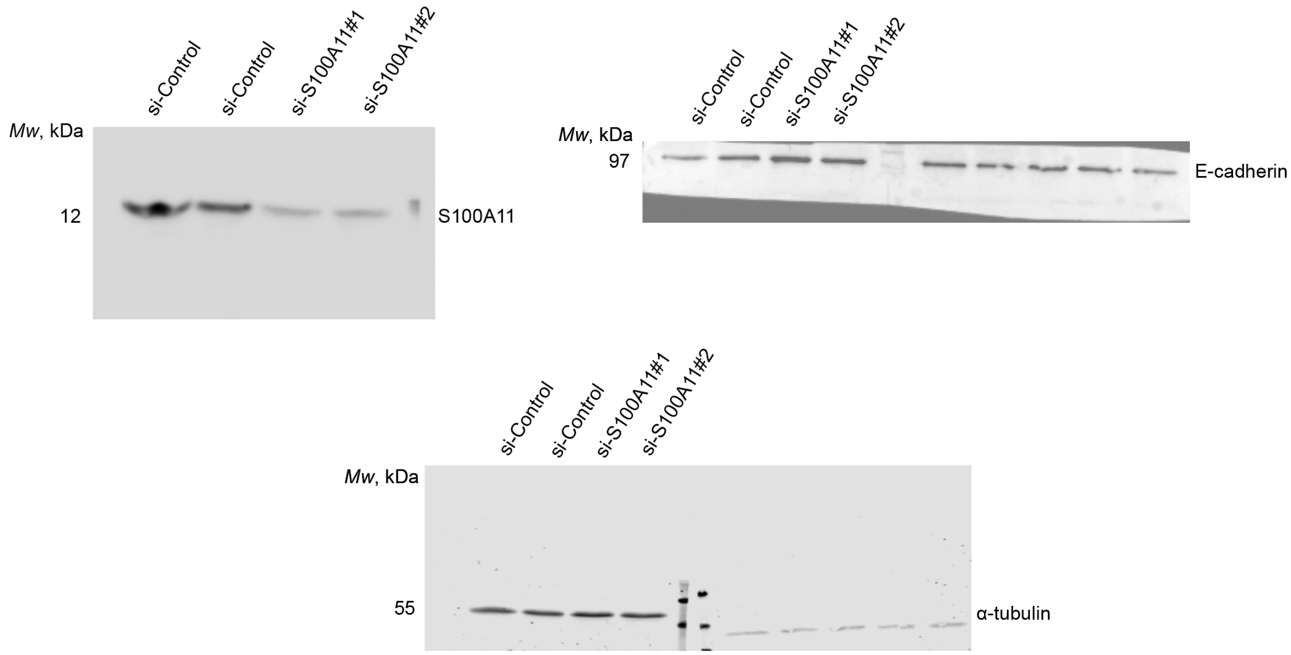

Fig. S5. Blot Transparency. Images of the uncropped blots with molecular weight markers (in kDa) are shown for all corresponding figures and supplementary figures.

Journal of Cell Science • Supplementary information

Figure 6A

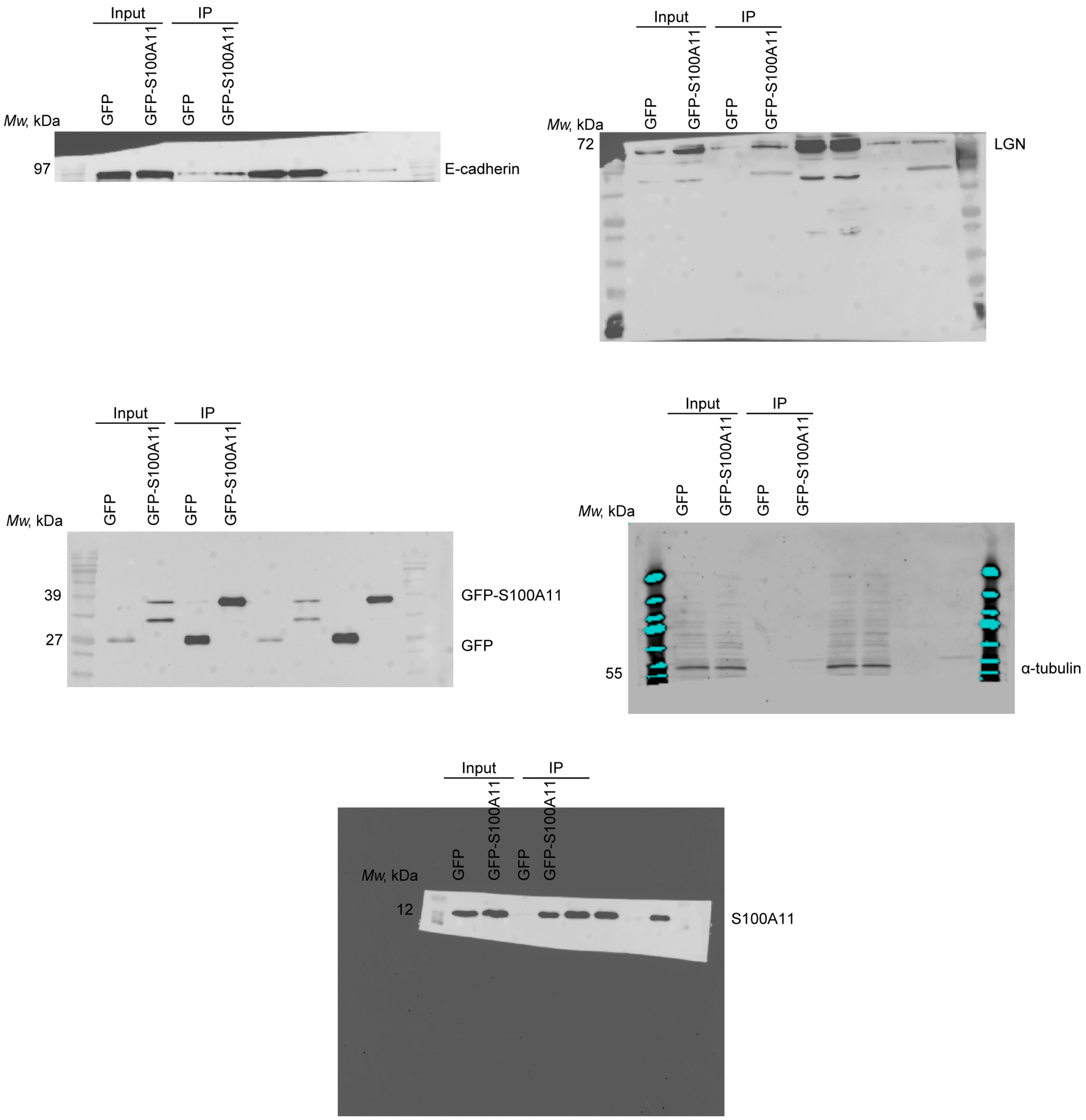
Input
IP
GFP
GFP-S100A11
GFP
GFP-S100A11
Mw, kDa
97
E-cadherin
Input
IP
GFP
GFP-S100A11
GFP
GFP-S100A11
Mw, kDa
72
LGN
Input
IP
GFP
GFP-S100A11
GFP
GFP-S100A11
Mw, kDa
39
27
GFP-S100A11
GFP
Input
IP
GFP
GFP-S100A11
GFP
GFP-S100A11
Mw, kDa
55
α-tubulin
Input
IP
GFP
GFP-S100A11
GFP
GFP-S100A11
Mw, kDa
12
S100A11

Figure 6H

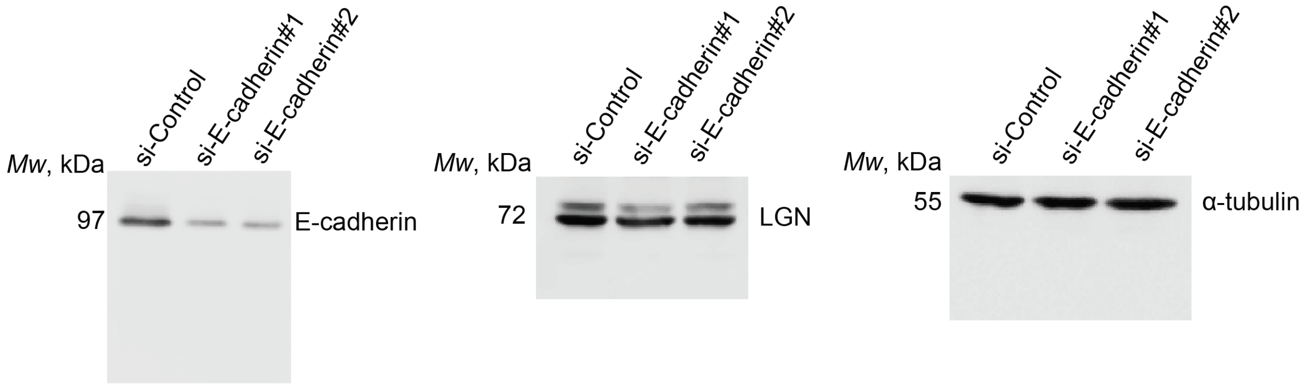

Figure S4B

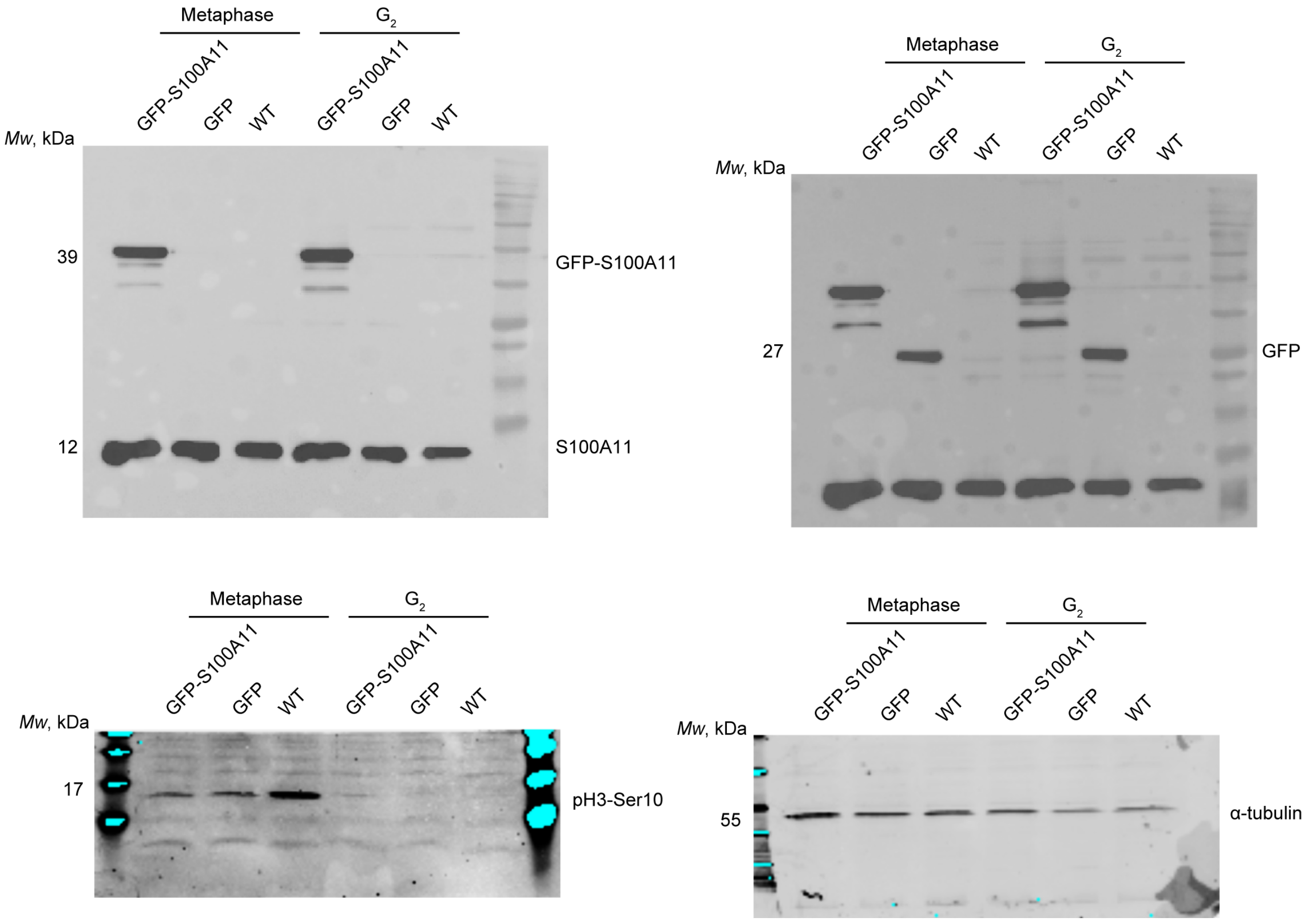

Table S1.

Available for download at
https://journals.biologists.com/jcs/article-lookup/doi/10.1242/jcs.261701#supplementary-data

Journal of Cell Science • Supplementary information

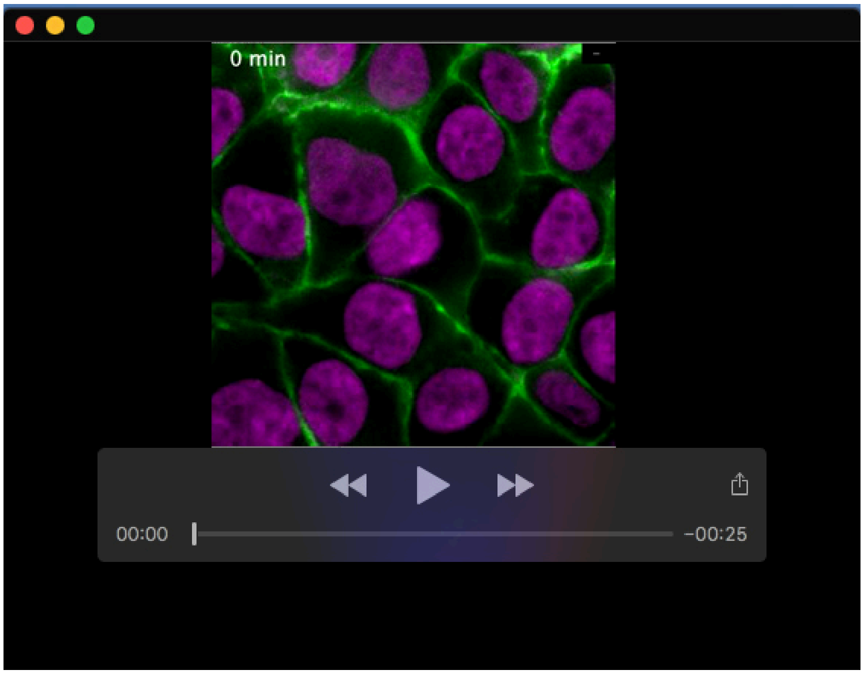

Movie 1. Description: CellMask^{TM} circumferential distribution at the plasma membrane and correct mitosis dynamic progression and outcome in polarised MCF-10A cells. Maximum intensity projections of CellMask^{TM} (green) and Hoechst (DNA, magenta) are shown through time (min).

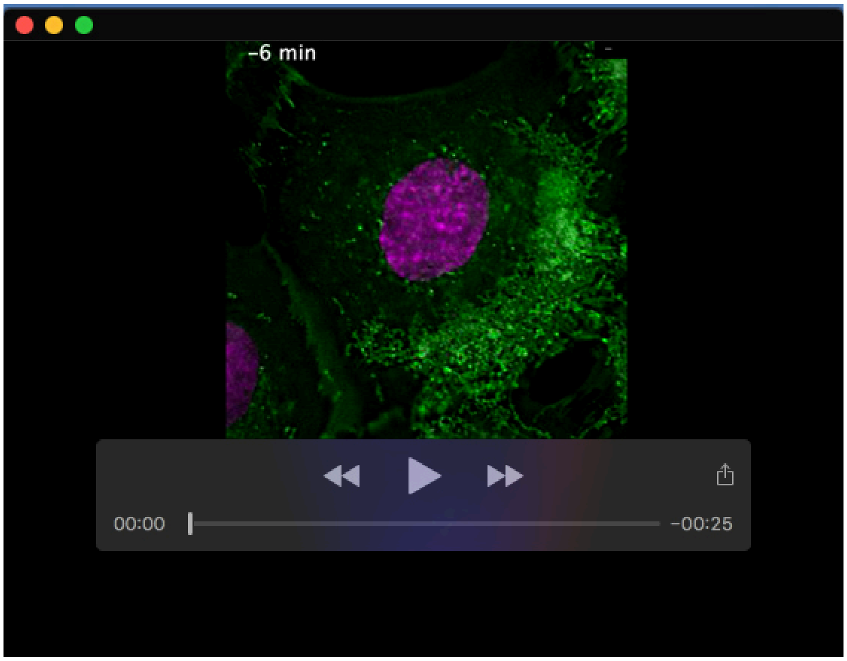

Movie 2. Description: Perturbation of cell-cell adhesion formation results in unilateral distribution of CellMask^{TM} and asymmetric elongation of the plasma membrane and impairs mitosis dynamic progression and generates unequal-sized daughter cells in non-polarised MCF-10A cells. Maximum intensity projections of CellMask^{TM} (green) and Hoechst (DNA, magenta) are shown through time (min).

Journal of Cell Science • Supplementary information

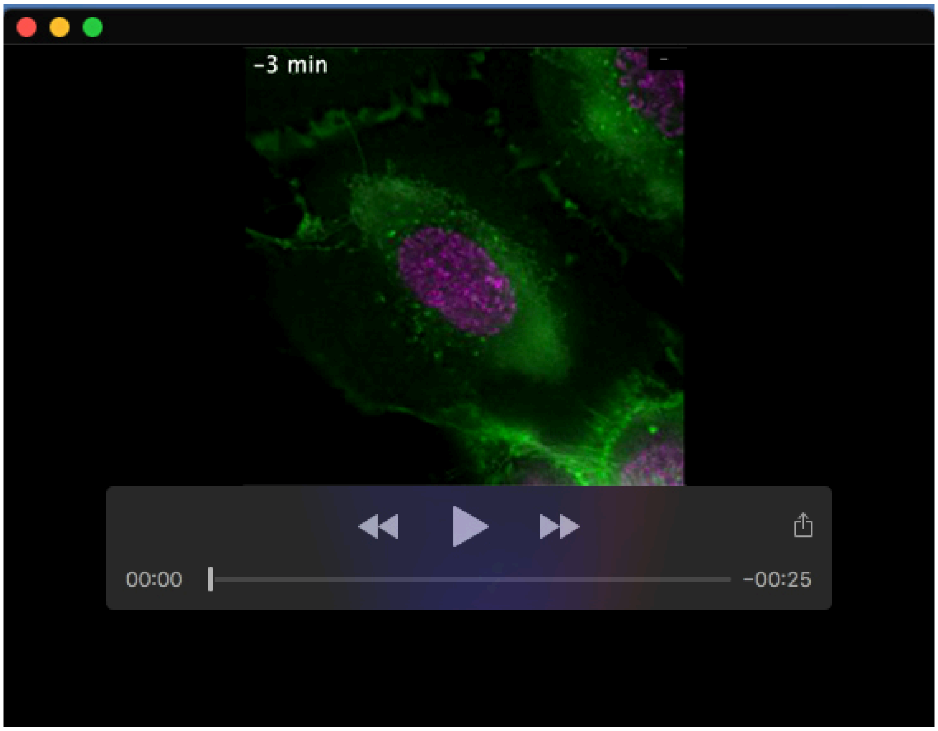

Movie 3. Description: Perturbation of cell-cell adhesion formation results in bilateral distribution of CellMask^{TM} and at the plasma membrane and delayed mitosis progression in non-polarised MCF-10A cells. Maximum intensity projections of CellMask^{TM} (green) and Hoechst (DNA, magenta) are shown through time (min).

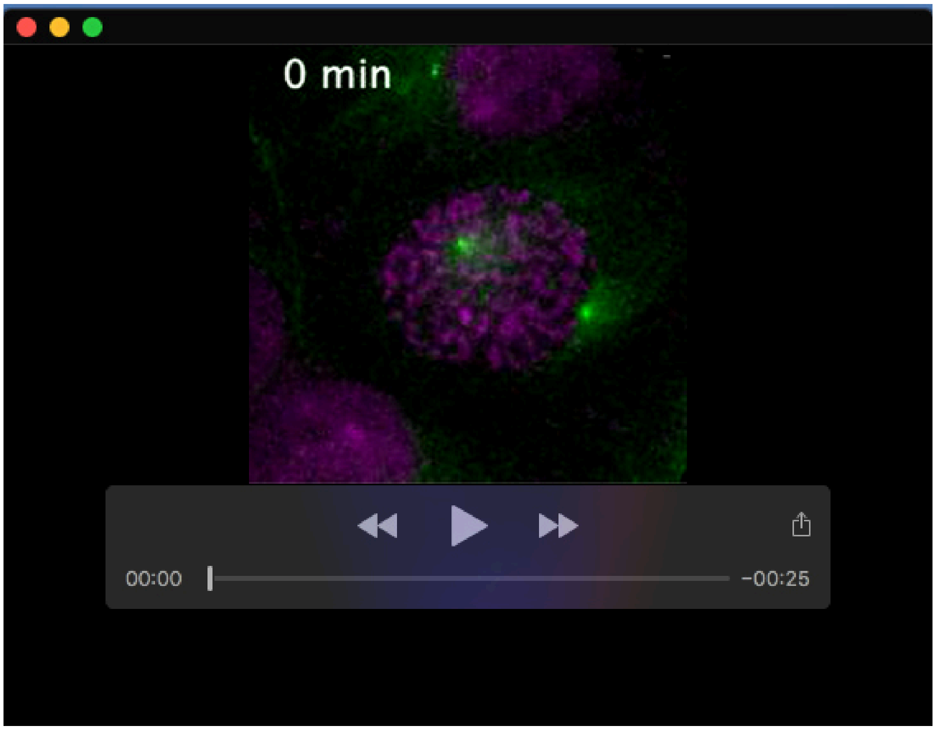

Movie 4. Description: Correct mitotic spindle and chromosome dynamics and mitosis progression and outcome in polarised MCF-10A cells. Maximum intensity projections of SiR-tubulin (green) and Hoechst (DNA, magenta) are shown through time (min).

Journal of Cell Science • Supplementary information

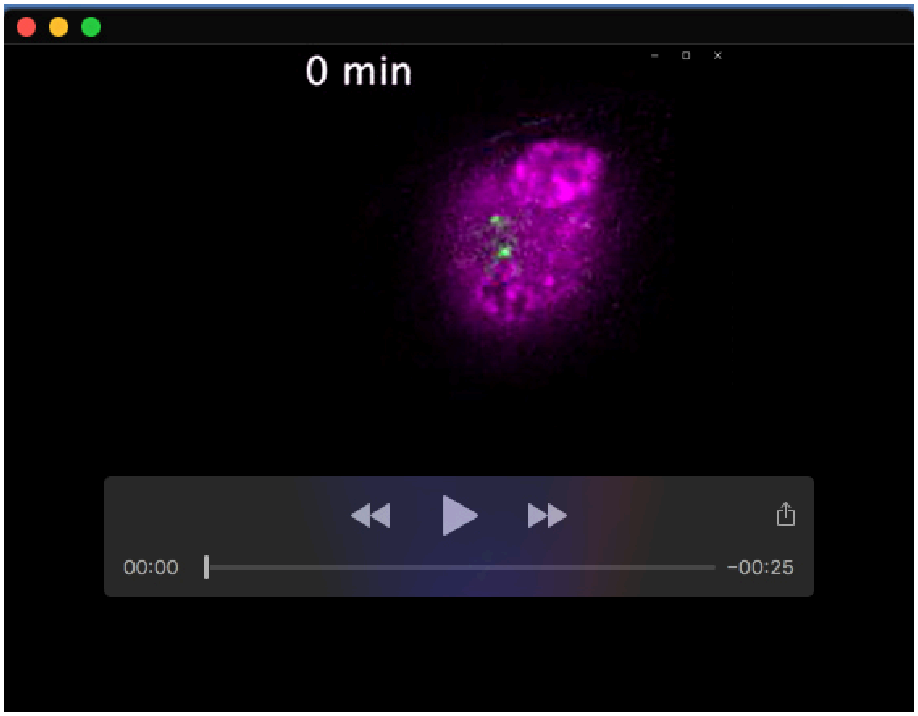

Movie 5. Description: Perturbation of cell-cell adhesion formation results in chromosome mis-segregation, micronuclei, and delayed mitosis progression in non-polarised MCF-10A cells. Maximum intensity projections of SiR-tubulin (green) and Hoechst (DNA, magenta) are shown through time (min).

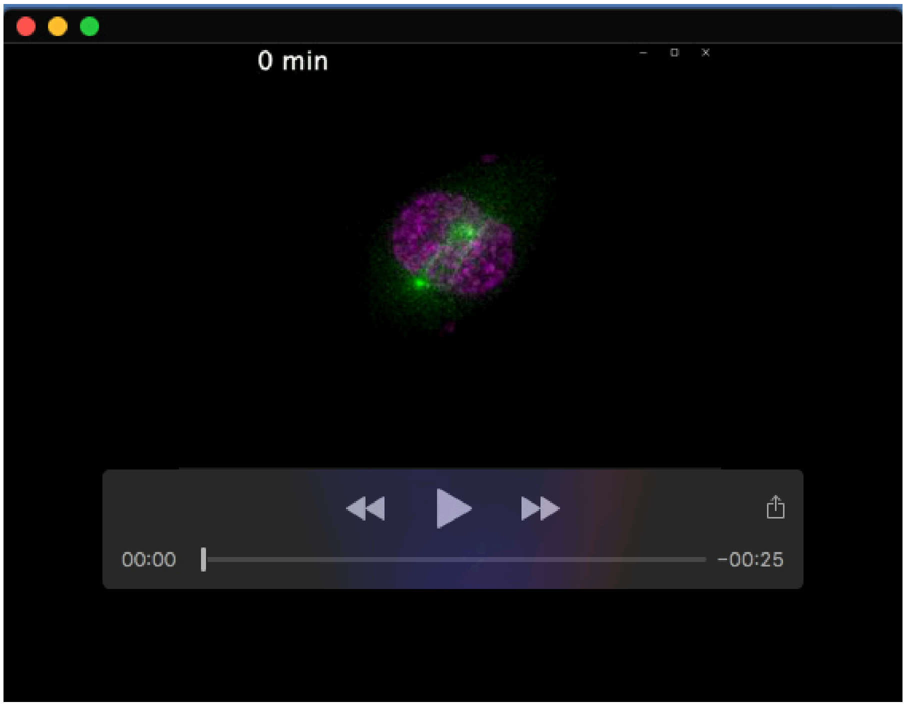

Movie 6. Description: Perturbation of cell-cell adhesion formation results in chromosome bridges and delayed mitosis progression in non-polarised MCF-10A cells. Maximum intensity projections of SiR-tubulin (green) and Hoechst (DNA, magenta) are shown through time (min).

Journal of Cell Science • Supplementary information

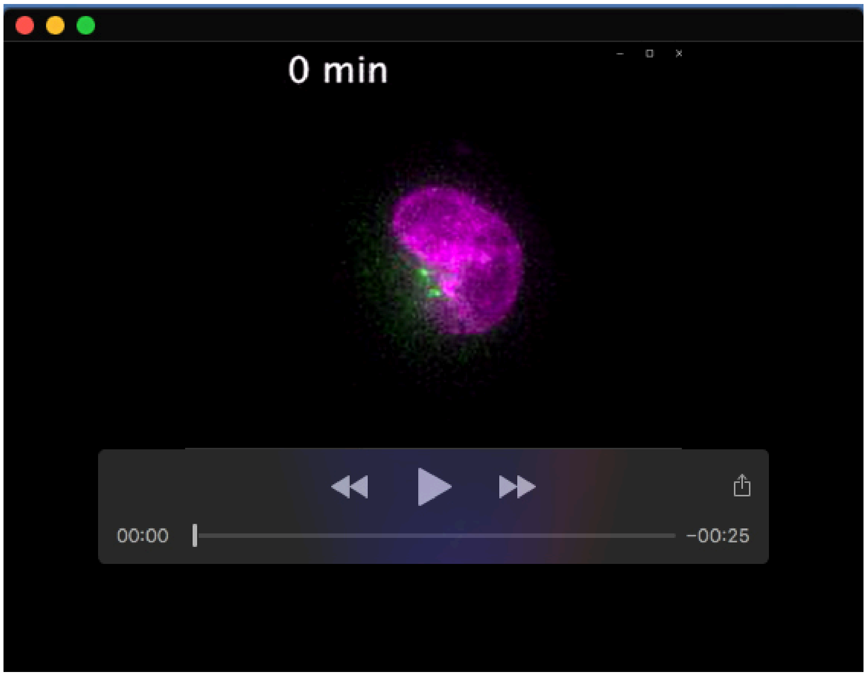

Movie 7. Description: Perturbation of cell-cell adhesion formation results in mitotic spindle dynamic and assembly defects, and mitotic arrest in non-polarised MCF-10A cells. Maximum intensity projections of SiR-tubulin (green) and Hoechst (DNA, magenta) are shown through time (min).

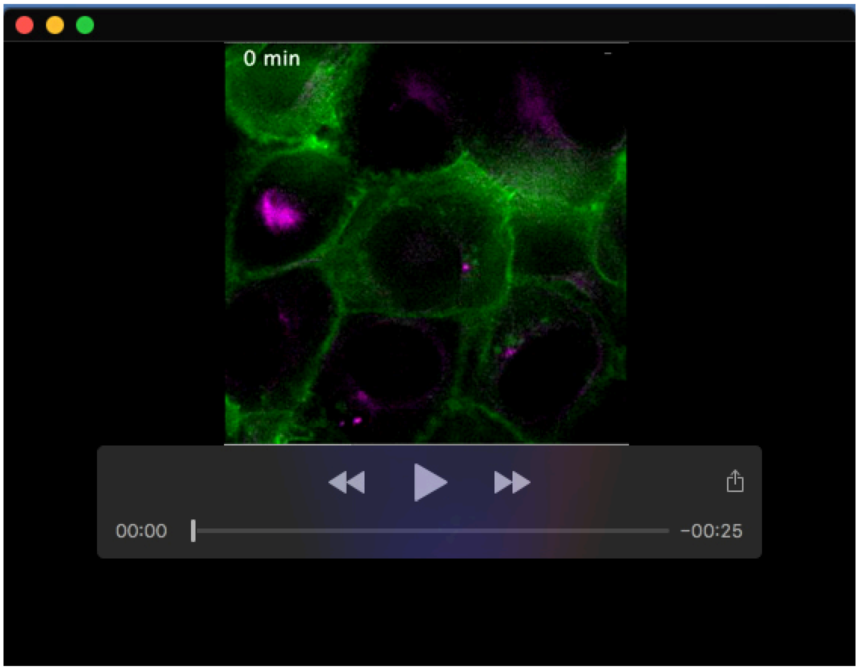

Movie 8. Description: Correct F-actin dynamic re-organisation and mitotic spindle dynamics and mitosis progression in polarised MCF-10A cells stably expressing Lifeact-mCherry. Maximum intensity projections of Lifeact-mCherry (F-actin, green) and SiR-tubulin (magenta) are shown through time (min).

Journal of Cell Science • Supplementary information

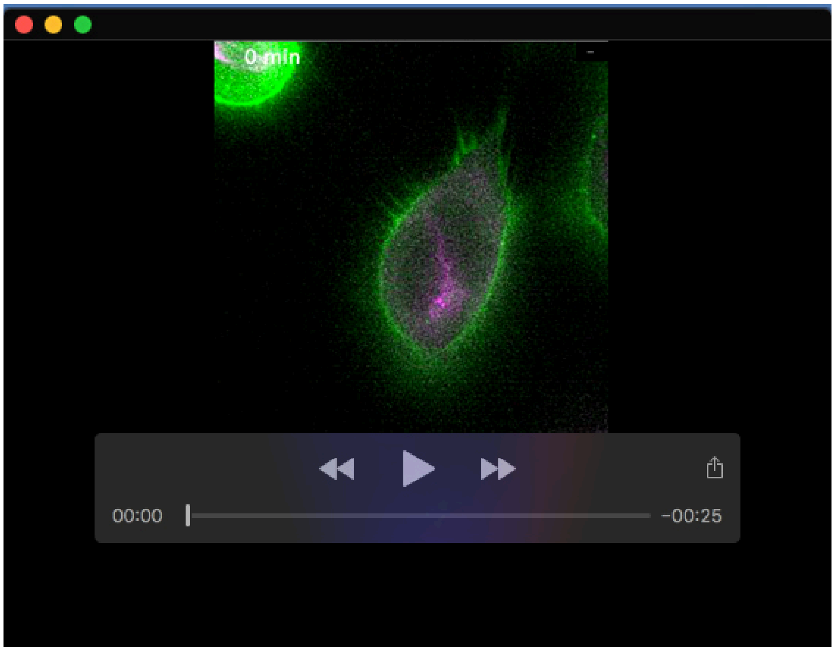

Movie 9. Description: Perturbation of cell-cell adhesion formation affects F-actin re-organisation and results in mitotic spindle assembly and dynamic defects and impairs mitosis progression and outcome in non-polarised MCF-10A cells stably expressing Lifeact-mCherry. Maximum intensity projections of Lifeact-mCherry (F-actin, green) and SiR-tubulin (magenta) are shown through time (min).

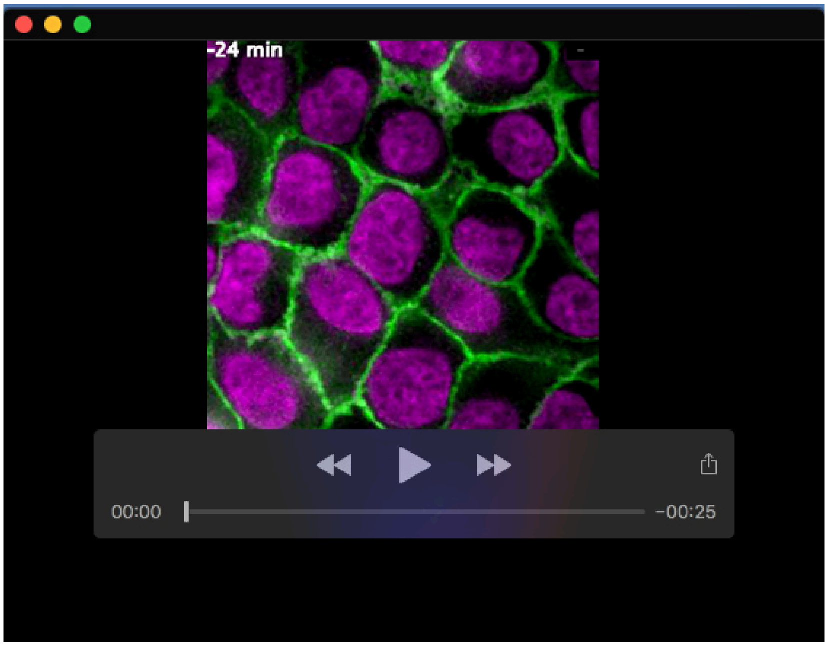

Movie 10. Description: CellMask™ circumferential distribution at the plasma membrane and correct dynamics of mitosis in polarised MCF-10A cells treated with si-Control. Maximum intensity projections of CellMask™ (green) and Hoechst (DNA, magenta) are shown through time (min).

Journal of Cell Science • Supplementary information

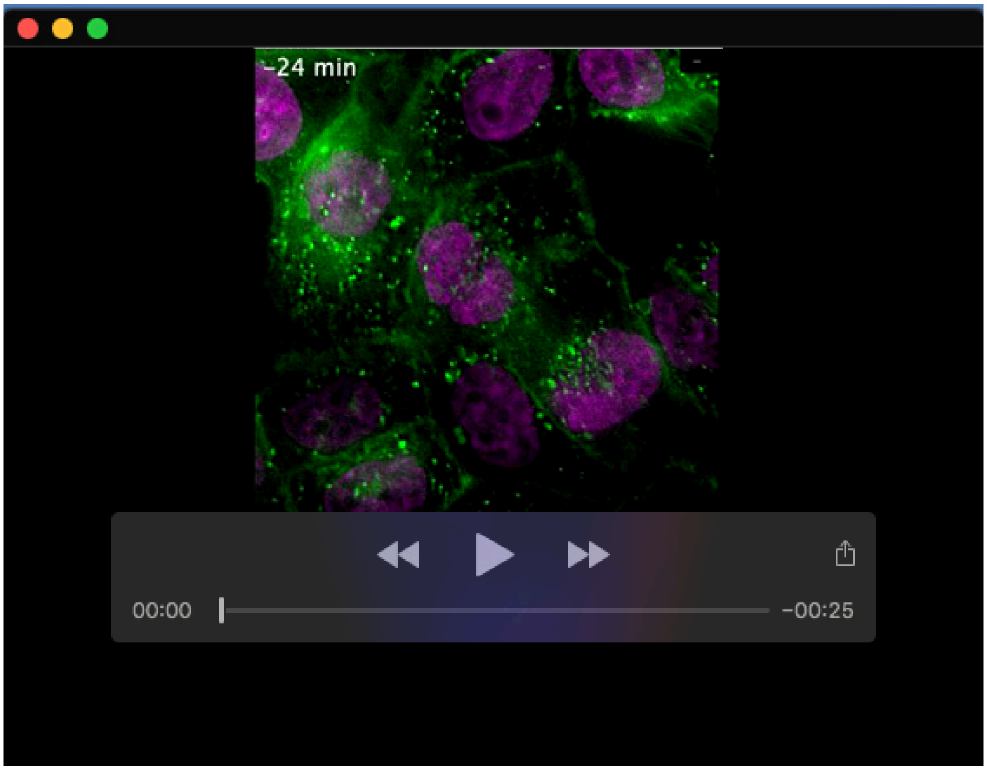

Movie 11. Description: S100A11 knockdown (si-S100A11#1) in polarised MCF-10A cells results in unilateral distribution of CellMask™ and asymmetric elongation of the plasma membrane and impairs mitosis dynamic progression and outcome. Maximum intensity projections of CellMask™ (green) and Hoechst (DNA, magenta) are shown through time (min).

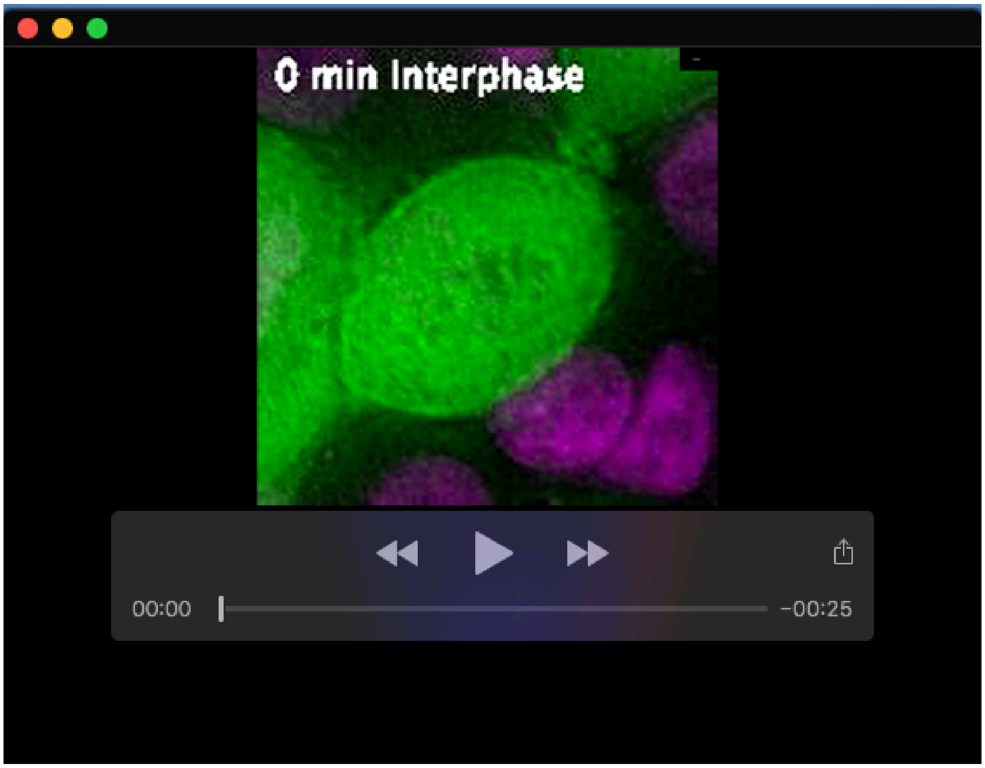

Movie 12. Description: S100A11 spatiotemporal distribution in polarised MCF-10A cells stably expressing GFP-S100A11. Maximum intensity projections of GFP-S100A11 (green) and Hoechst (DNA, magenta) are shown through mitosis phases.

Journal of Cell Science • Supplementary information
