# Supplementary material for: Interplay between the plasma membrane and cell–cell adhesion maintains epithelial identity for correct polarised cell divisions
Source: J Cell Sci. 2023 Nov 28;137(5):jcs261701. doi: 10.1242/jcs.261701 (PMC10729819; doi:10.1242/jcs.261701)
Supplement: Supplementary file 1 [file joces-137-261701_review_history.pdf]

Interplay between the plasma membrane and cell-cell adhesion maintains epithelial identity for correct polarised cell divisions

Manal M Hosawi, Jiaoqi Cheng, Maria Fankhaenel, Marcin R Przewloka and Salah Elias
DOI: 10.1242/jcs.261701

Editor: David Bryant

Review timeline
Submission to Review Commons: 20 May 2023
Original submission: 3 October 2023
Accepted: 17 October 2023

Reviewer 1

Evidence, reproducibility and clarity

The manuscript "Crosstalk between the plasma membrane and cell-cell adhesion maintains epithelial identity for correct polarised cell divisions" by Dr. Hosawi and colleagues reports the characterisation of the mitotic connection between plasma membrane dynamics and division orientation in polarised mammalian epithelial cells in culture. The authors start from the comparison of mitotic events of human mammary MCF10A cells grown at optimal density or at low density. They observed that only at optimal density MCF10A cells polarise by E- cadherin mediated cell-cell contacts, and display uniform membrane enrichment at the cortex, whereas cells grown at low density do not show cortical E-Cadherin enrichment, and distribute aberrantly the plasma membrane at one side and in cytoplasmic vesicles, generating daughter cells with unequal size. Consistently, further analyses revealed that low- density MCF10A cells undergo misoriented mitosis, with chromosome congression and misegregetion defects. Mechanistically, low density MCF10A cells fail to organise a symmetric mitotic spindle and center it in metaphase. This is due to an increased cortical actomyosin thickness coupled to abnormal astral microtubule stability. Building on previous data from the Elias lab, the authors uncover a role of the membrane-associated S100A11 protein in maintaining correct plasma membrane dynamics and E-cadherin localisation in mitosis. Further dissection of the molecular mechanism underlying this mitotic function od S10011A revealed that it enriches at the cortex only in optimal-density MCF10A cells, and promotes spindle orientation by association with LGN and E-cadherin, upstream of E- cadherin. This evidence depicts the plasma membrane and S100A11 proteins as a key mechanical sensors of cell-cell adhesion orchestrating the recruitment of E-cadherin and LGN-dependent force generators to ensure correct division orientation.

Major points:

- Important information is presented in Supplementary Figure S3. I suggest to move these panels in the main figures. Specifically, I would replace figure 4A with S3A showing the distribution of endogenous S100A11 in MCF10A cells, rather than the one of the GFP-tagged version which is over-expressed.
- The mechanisms of division orientation governed by S100A11 seems to impinge on the control of cortical F-actin and astral microtubule dynamics. This is illustrated in figure S3C, which in my opinion should be shown in the main figures with some more explanation / experiments. The authors mention the " tight actin F-actin bundles at the cell-cell contacts" that are lost in S100A11-depleted cells, and that interact with astral microtubules. However this is not fully clear in figure S3C. I think the authors should find a way to present better these evidence which is key in supporting their molecular model.
- I think the discussion would benefit from the addition of a graphical cartoon model

© 2023. Published by The Company of Biologists under the terms of the Creative Commons Attribution License (https://creativecommons.org/licenses/by/4.0/).

illustrating the role of S100A11 in controlling plasma membrane dynamics in mitosis and spindle orientation.
- Finally, to understand the relevance of S100A11 in the context of 3D polarised mammary epithelia, it would be very interesting to analyse the effect of S100A11 knock-downn in mouse mammary epithelial acini grown in matrigel. This is not essential for the proposed studies, but would add biological relevance to the mechanisms characterised in 2D colture.

Minor comments:

- It would be preferable to mention the known functions of S100A11 in the introduction rather than at the beginning of the paragraph at pg. 9.
- at pg 10, beginning of paragraph, I find it a weird phrasing that "LGN interacts with F-actin". As reported in the reference cited here, this is through Afadin, which binds simultaneously LGN and cortical F-actin. I would rephrase it.

Significance

The description of cell adhesion as key factor instructing correct mitotic progression and execution of oriented division of vertebrate epithelial cells by controlling plasma membrane dynamics is novel and interesting for scientist in the spindle orientation/polarity field. The experiments are well-designed and perfectly executed and presented. I am in favour of publication of the manuscript, providing that a few points are addressed.

Reviewer 2

Evidence, reproducibility and clarity

Establishment and maintenance of cell polarity are fundamental processes for physiology in multi-cellular organism given the fact that more than 380 million epithelial cell renewal for every second in human adults. However, the precise mechanisms linking plasma membrane polarity and cortical cytoskeleton dynamics of epithelial cells during mitotic exit and interphase remain ill-illustrated. Salah Elias and her colleagues experimentally manipulated the density of mammary epithelial cells in culture, which led to several mitotic defects.
Specifically, they found that perturbation of cell-cell adhesion integrity impairs the dynamics of the plasma membrane during mitosis, affecting the shape and size of mitotic cells and resulting in defects in mitosis progression and generating daughter cells with aberrant cytoarchitecture. In these conditions, F-actin-astral microtubule crosstalk is impaired leading to mitotic spindle misassembly and misorientation, which in turn contributes to chromosome mis-segregation. Mechanistically, they identified the S100 Ca2+-binding protein A11 as a key membrane-associated regulator that forms a complex with E-cadherin and LGN to coordinate plasma membrane remodelling with E-cadherin-mediated cell adhesion and LGN- dependent mitotic spindle machinery. I felt that this is a strong manuscript for peer-review as it serves diversified interests in modern cell biology.

Significance

Several key cellular experiments should be repeated using a second line of epithelial cells such as RPE1.

Reviewer 3

Evidence, reproducibility and clarity

Summary: your understanding of the study and its conclusions.

The scope of the study is to understand the links between cell-cell adhesion integrity, plasma membrane dynamics and mitotic spindle in mammalian epithelial tissues. To test this, the authors cultured mammary epithelial cells at optimal or low density as a way of perturbing cell-cell

© 2023. Published by The Company of Biologists under the terms of the Creative Commons Attribution License (https://creativecommons.org/licenses/by/4.0/).

adhesion. The authors conclude that perturbing cell-cell adhesion alters plasma membrane dynamics, causing mitotic defects and that S100A11 coordinates this link via E- cadherin. Whilst this is an interesting manuscript, illustrating the differences of mitotic success in optimal density vs. low density cell cultures, I do not think that the conclusions are supported by the evidence presented for the reasons stated below.

Major comments: major issues affecting the conclusions.

The manuscript clearly shows that culturing cells at a lower density results in a higher incidence of asymmetric division (figure 1) and mitosis defects (figure 2). Cells round more and faster and there is more actin at the cortex during rounding (figure 3). However, whilst differences in cell-cell adhesion are likely to play a role in mediating these effects, I don't think that it is possible to claim from the data presented that these defects are specifically due to cell-cell adhesion differences. This is because the morphology of cells at low density is also very different - cells appear more mesenchymal, with migratory front-rear polarity instead of apical-basal polarity. These cells will therefore have many differences between them, cell-adhesion being just one. The data is also not showing a 'loss' of cell-cell adhesion integrity but are rather illustrating the differences between cells that have formed cell-cell adhesions and those that have not. To really test the specific role of cell-cell adhesions, the authors would need to inhibit adhesions directly but without altering the cell density - for example via chemical or genetic perturbation within a confined environment. I suggest that the authors either need to do these experiments or to requalify what their data is telling us. The current manuscript also demonstrates that cell adhesion is affected when S100A11 is knocked down (figure 4). It shows binding between and colocalization of S100A11 and E- cadherin, and shows that LGN cortical distribution is affected when S100A11 is knocked down (Figure 5). The results presented are suggestive of S100A11 being upstream of E- cadherin. However, I don't understand how the data shows "crosstalk between the plasma membrane, cell-cell adhesion, and the cell cortex during mitosis". For example, on P9: "We observed unequal distribution of CellMaskTM in a vast majority of S100A11-depleted cells (si-S100A11#1: ~79% versus si-Control: ~26%), indicating defects in plasma membrane remodelling (Figures 4B and 4C)." I don't agree that this demonstrates a defect in PM remodelling. Rather the cells in the representative images are less adherent and have adopted a more migratory cell state similar to that seen in figure 1 when seeded at low density. The fluidity of the much larger cells shown in knock down cells in panel F also appears higher, again suggesting an adhesion defect. An earlier paper from the same lab this year identified Annexin A1 as directing mitotic spindle orientation via localising LGN at lateral cortex. During this earlier paper they also identified S100A11, which is a partner for Annexin A1. The authors could more clearly explain what S100A11 is in the current manuscript and how the current study builds on this earlier study.

Based on the data presented, I suggest that the authors should requalify their data. I suggest that the conclusions that can be drawn from the data are that cellular state is important for regulating mitosis orientation and fidelity (i.e. adherent epithelia cells vs. less adherent more migratory cells). S100A11 is important for promoting cell-cell adhesions and might be upstream of the known role of E-cadherin in regulating spindle orientation. Whilst I suggest that more quantified experiments would need to be included in order to assess possible effects on plasma membrane remodelling, the manuscript could be generally improved by a clearer explanation of the open question that they are addressing and what specific advance this manuscript has made in relation to the current literature, including their own. I do not currently feel that the title of the manuscript is appropriate since I don't think that a crosstalk between the plasma membrane and cell-cell adhesion has been shown here.

Minor comments: important issues that can confidently be addressed.

P3: I wouldn't describe the junctional proteins listed as polarity proteins.
Figure 1 - can the membrane blebbing phenotype by quantified? At the moment this part is observational so can't really be used to determine the role of plasma membrane remodelling.

Figure 3. I'm not sure what the 'subcortical actin cloud' measurement is. Figure 3G suggests it may be the distance from the cortex to the spindle pole but how does this relate to actin?

Figure 4A. I can't see GFP-S100A11 accumulating at the cell surface. To me these images suggest

© 2023. Published by The Company of Biologists under the terms of the Creative Commons Attribution License (https://creativecommons.org/licenses/by/4.0/).

that it is relatively ubiquitously expressed throughout the cytoplasm and surface, which is different to the later antibody stains, that show localisation at the cell surface.

Fig 4H doesn't show an active process of translocation of E-Cadherin to the cytoplasm. It shows representative images with slightly higher levels of E-Cadherin in the cytoplasm. This could be due to translocation or it could be to do with lack of E-Cadherin assembly.

4I I don't understand where the line profile is derived from - where is apical and where is basal in the images? Could a diagram be included?

The discussion could be shortened and more clearly written - perhaps with subheadings of the main findings.

Methods: Why is cholera toxin used in the cell culture medium?

Significance

In general, this is an interesting paper about the fidelity of mitosis in cells in adherent monolayers vs. in more migratory, non-adherent states. There is existing literature on this topic (some cited in the manuscript, alongside reviews of the topic).

The main conceptual advance, as far as I can see, is that S100A11 is important for promoting cell-cell adhesions and might be upstream of the known role of E-cadherin in regulating spindle orientation via LGN. The main limitation is that plating cells at different densities is not a direct 'perturbation' of cell-cell adhesion. This means that the phenotypes seen could be due to many factors, not just cell adhesion. Assessment of plasma membrane and cytoskeletal dynamics are also often observational and not conclusive.

The manuscript would be of interest to basic researchers working on epithelial development. Also potentially to basic researchers working on cancer, due to the mitotic errors described.

I have expertise in epithelial cell biology.

I estimate the authors would need between 3 and 6 months for revisions if they decide to do further experiments and between 1 and 3 months if they decide to re-qualify their claims.

<u>**Author response to reviewers' comments**</u>

Manuscript number: RC-2023-02013
Corresponding author(s):
Salah Elias
Marcin Przewloka

1. General Statements [optional]

We would like to thank Review Commons for their innovative approach to scientific peer-review and publishing. We thank all the Reviewers for their positive, highly complementary assessment of the manuscript and for highlighting the high quality and reproducibility of the work and the novelty and significance of the results: *“The experiments are well-designed and perfectly executed and presented”; “I felt that this is a strong manuscript for peer-review as it serves diversified interests in modern cell biology.”; “The manuscript would be of interest to basic researchers working on epithelial development. Also potentially to basic researchers working on cancer, due to the mitotic errors described.”*. We are grateful for the Reviewers’ comments and suggestions that have contributed to improving the revised manuscript. We have addressed all the Reviewers’ concerns, as detailed below in the point-by-point response to the Reviewers. Textual changes in the revised manuscript are marked in Blue.

© 2023. Published by The Company of Biologists under the terms of the Creative Commons Attribution License (https://creativecommons.org/licenses/by/4.0/).

2. Point-by-point description of the revisions

Reviewer #1 (Evidence, reproducibility and clarity (Required)):

The manuscript "Crosstalk between the plasma membrane and cell-cell adhesion maintains epithelial identity for correct polarised cell divisions" by Dr. Hosawi and colleagues reports the characterisation of the mitotic connection between plasma membrane dynamics and division orientation in polarised mammalian epithelial cells in culture. The authors start from the comparison of mitotic events of human mammary MCF10A cells grown at optimal density or at low density. They observed that only at optimal density MCF10A cells polarise by E-cadherin mediated cell-cell contacts, and display uniform membrane enrichment at the cortex, whereas cells grown at low density do not show cortical E-Cadherin enrichment, and distribute aberrantly the plasma membrane at one side and in cytoplasmic vesicles, generating daughter cells with unequal size. Consistently, further analyses revealed that low-density MCF10A cells undergo misoriented mitosis, with chromosome congression and misegregetion defects. Mechanistically, low density MCF10A cells fail to organise a symmetric mitotic spindle and center it in metaphase. This is due to an increased cortical actomyosin thickness coupled to abnormal astral microtubule stability. Building on previous data from the Elias lab, the authors uncover a role of the membrane-associated S100A11 protein in maintaining correct plasma membrane dynamics and E-cadherin localisation in mitosis. Further dissection of the molecular mechanism underlying this mitotic function od S10011A revealed that it enriches at the cortex only in optimal-density MCF10A cells, and promotes spindle orientation by association with LGN and E-cadherin, upstream of E-cadherin. This evidence depicts the plasma membrane and S100A11 proteins as a key mechanical sensors of cell-cell adhesion orchestrating the recruitment of E-cadherin and LGN- dependent force generators to ensure correct division orientation.

Major points:
- Important information is presented in Supplementary Figure S3. I suggest to move these panels in the main figures. Specifically, I would replace figure 4A with S3A showing the distribution of endogenous S100A11 in MCF10A cells, rather than the one of the GFP-tagged version which is over-expressed.

Authors response: We thank the Reviewer for this suggestion. We have now moved Figure S3A to Figure 4, to replace Figure 4A and show the localisation of endogenous S100A11 during mitosis and included new quantifications in new Figure 4B. We have moved Figure 3A to supplementary figures (new Figure S4A). We have amended the text of the results section and the Source Data file accordingly.

- The mechanisms of division orientation governed by S100A11 seems to impinge on the control of cortical F-actin and astral microtubule dynamics. This is illustrated in figure S3C, which in my opinion should be shown in the main figures with some more explanation / experiments. The authors mention the " tight actin F-actin bundles at the cell-cell contacts" that are lost in S100A11-depleted cells, and that interact with astral microtubules. However this is not fully clear in figure S3C. I think the authors should find a way to present better these evidence which is key in supporting their molecular model.

Authors response: As requested by the Reviewer we have now moved Figure S3C to the main manuscript, as new Figure 5. To clarify further the effect of S100A11 depletion on the tight actin bundle formation at the cell-cell contacts, we have now included a new illustration in new Figure 5C. Additionally, we have clarified further these findings in the results section (page 11). While we agree with the Reviewer that additional experiments, for example using live imaging of MCF-10A cells co-labelled for F-actin and tubulin, would help assess further the crosstalk between cortical actin and astral microtubules, based on our experience these live imaging experiments are challenging and can take up to several months to optimise and may not warrant successful outcome.

- I think the discussion would benefit from the addition of a graphical cartoon model illustrating the role of S100A11 in controlling plasma membrane dynamics in mitosis and spindle orientation.

Authors response: We thank the Reviewer for this suggestion. We have now added a graphical

© 2023. Published by The Company of Biologists under the terms of the Creative Commons Attribution License (https://creativecommons.org/licenses/by/4.0/).

cartoon (new Figure 7), summarising the role of S100A11-mediated regulation of plasma membrane dynamics in polarised cell division orientation, progression and outcome. We hope this new illustration clarifies further the mechanisms described in this study.

- Finally, to understand the relevance of S100A11 in the context of 3D polarised mammary epithelia, it would be very interesting to analyse the effect of S100A11 knock-downn in mouse mammary epithelial acini grown in matrigel. This is not essential for the proposed studies, but would add biological relevance to the mechanisms characterised in 2D colture.

Authors response: We agree with the Reviewer that validating our findings in 3D cultures of mammary epithelial cells will be important to determine the influence of S100A11-mediated regulation of plasma membrane dynamics during mitosis on lumen formation and tissue morphogenesis. This is exactly the direction where the follow-up of these findings will go. While the first author who led this work has graduated and left our lab, we have recently recruited a new PhD student to address this important question, which will need a few years of investigation to provide important insights, similarly to what we did in our previous work (Fankhaenel *et al.*, 2023 *Nat Commun*).

Minor comments:

- It would be preferable to mention the known functions of S100A11 in the introduction rather than at the beginning of the paragraph at pg. 9.

Authors response: In response to the Reviewer's suggestion, we have now moved the paragraph describing known functions of S100A11 to the introduction of the revised manuscript (see page 5).

- at pg 10, beginning of paragraph, I find it a weird phrasing that "LGN interacts with F-actin". As reported in the reference cited here, this is through Afadin, which binds simultaneously LGN and cortical F-actin. I would rephrase it.

Authors response: We thank the Reviewer for clarifying this point, which we have now rectified in the revised manuscript (see page 11).

Reviewer #1 (Significance (Required)):

The description of cell adhesion as key factor instructing correct mitotic progression and execution of oriented division of vertebrate epithelial cells by controlling plasma membrane dynamics is novel and interesting for scientist in the spindle orientation/polarity field. The experiments are well- designed and perfectly executed and presented. I am in favour of publication of the manuscript, providing that a few points are addressed.

Authors response: We thank the Reviewer for their very positive evaluation of our work.

Reviewer #2 (Evidence, reproducibility and clarity (Required)):

Establishment and maintenance of cell polarity are fundamental processes for physiology in multi-cellular organism given the fact that more than 380 million epithelial cell renewal for every second in human adults. However, the precise mechanisms linking plasma membrane polarity and cortical cytoskeleton dynamics of epithelial cells during mitotic exit and interphase remain ill-illustrated. Salah Elias and her colleagues experimentally manipulated the density of mammary epithelial cells in culture, which led to several mitotic defects. Specifically, they found that perturbation of cell-cell adhesion integrity impairs the dynamics of the plasma membrane during mitosis, affecting the shape and size of mitotic cells and resulting in defects in mitosis progression and generating daughter cells with aberrant cytoarchitecture. In these conditions, F-actin-astral microtubule crosstalk is impaired leading to mitotic spindle misassembly and misorientation, which in turn contributes to chromosome mis-segregation. Mechanistically, they identified the S100 Ca2+-binding protein A11 as a key membrane-associated regulator that forms a complex with E-cadherin and LGN to coordinate plasma membrane remodelling with E-cadherin-mediated

© 2023. Published by The Company of Biologists under the terms of the Creative Commons Attribution License (https://creativecommons.org/licenses/by/4.0/).

cell adhesion and LGN-dependent mitotic spindle machinery. I felt that this is a strong manuscript for peer-review as it serves diversified interests in modern cell biology.

Authors response: We thank the Reviewer for their overall very positive feedback on our manuscript.

Reviewer #2 (Significance (Required)):

Several key cellular experiments should be repeated using a second line of epithelial cells such as RPE1.

Authors response: We agree with the Reviewer it will be interesting to test our findings in other epithelial cells, including RPE1 cells, a widely used epithelial cell model to study the mechanisms controlling cell division. Nonetheless, we would like to emphasise that while our work demonstrates the importance of the interplay between plasma membrane dynamics and cell-cell adhesion for correct execution of polarised cell divisions in mammary epithelial cells, our aim is not to generalise the role of these S100A11-mediated mechanisms. An elegant study has shown that the mechanisms controlling plasma membrane remodelling and elongation during mitosis to ensure correct positioning of the mitotic spindle and symmetric division differ between HeLa cells and RPE1 cells (Kiyomitsu and Cheeseman, 2013 *Cell*). Additional experiments in a second cell line will require a thorough characterisation of the expression and localisation of S100A11 during the cell cycle, as well as the use of extensive and time-consuming knockdown and imaging experiments over several months and may lead to different observations requiring further mechanistic investigation, which is beyond the initial scope of this study. Additionally, the PhD student who led this study has graduated and left the lab and presently we don't have capacity or resources to conduct these suggested experiments. Finally, to precisely address the Reviewer's concern, we have now amended the revised manuscript to make our statements more specific to mammary epithelial cells throughout the text.

Reviewer #3 (Evidence, reproducibility and clarity (Required)):

Summary: your understanding of the study and its conclusions.
The scope of the study is to understand the links between cell-cell adhesion integrity, plasma membrane dynamics and mitotic spindle in mammalian epithelial tissues. To test this, the authors cultured mammary epithelial cells at optimal or low density as a way of perturbing cell-cell adhesion. The authors conclude that perturbing cell-cell adhesion alters plasma membrane dynamics, causing mitotic defects and that S100A11 coordinates this link via E-cadherin. Whilst this is an interesting manuscript, illustrating the differences of mitotic success in optimal density vs. low density cell cultures, I do not think that the conclusions are supported by the evidence presented for the reasons stated below.

Major comments: major issues affecting the conclusions.
- The manuscript clearly shows that culturing cells at a lower density results in a higher incidence of asymmetric division (figure 1) and mitosis defects (figure 2). Cells round more and faster and there is more actin at the cortex during rounding (figure 3). However, whilst differences in cell-cell adhesion are likely to play a role in mediating these effects, I don't think that it is possible to claim from the data presented that these defects are specifically due to cell-cell adhesion differences. This is because the morphology of cells at low density is also very different - cells appear more mesenchymal, with migratory front-rear polarity instead of apical-basal polarity. These cells will therefore have many differences between them, cell-adhesion being just one. The data is also not showing a 'loss' of cell-cell adhesion integrity but are rather illustrating the differences between cells that have formed cell-cell adhesions and those that have not. To really test the specific role of cell-cell adhesions, the authors would need to inhibit adhesions directly but without altering the cell density - for example via chemical or genetic perturbation within a confined environment. I suggest that the authors either need to do these experiments or to requalify what their data is telling us.

Authors response: We thank the Reviewer for their insightful discussion of the proposed mechanisms described in our manuscript. Several of the Reviewer's comments pinpoint and

© 2023. Published by The Company of Biologists under the terms of the Creative Commons Attribution License (https://creativecommons.org/licenses/by/4.0/).

exactly match the messages that we would like to convey to the scientific community. Therefore, to address the Reviewer's comments, we have carefully requalified our statements in several places in the revised manuscript, to ensure they are more clear and more precise.

We agree with the Reviewer's comment that our experiments using sub-optimal density of mammary epithelial cells rather prevents the formation of cell-cell adhesions than disturbing them. The Reviewer is right, our experiments in low-density cultures suggest that perturbation of cell-cell adhesion formation impairs mammary epithelial identity, where cells lose their polarity and adopt a more mesenchymal phenotype, associated with plasma membrane remodelling defects. This affects the dynamics and progression of cell division. Nonetheless, our observations suggest an interplay between cell-cell adhesion and the plasma membrane to maintains correct cell shape during mitosis. To test this hypothesis, we explored the function of S100A11 which we have identified in the LGN interactome in mitotic mammary epithelial cells (Fankhaenel *et al.*, 2023 *Nat Commun*), and which has been shown to interact with E-cadherin at adherens junctions in MDCK cells (Guo *et al.*, 2014 *Sci Signal*). This, together with the fact that S100A11 controls plasma membrane repair (Jaiswal *et al.*, 2014 *Nat Commun*), suggested S100A11 as an interesting candidate to investigate the interplay between cell-cell adhesion and membrane remodelling during mitosis. The data presented here suggest that we were right and the perturbation of our membrane-bound target, S100A11, indeed leads to the same mitotic phenotypes. S100A11 RNAi-mediated knockdown (48h) affects E-cadherin localisation at the plasma membrane and impairs cell-cell adhesion formation with effects on plasma membrane dynamics that phenocopy the defects observed in our low-density culture experiments. Remarkably, perturbation of cell-cell adhesions persisted in cell treated with si-S100A11 for 72h (see Figure S3). Of note, all our siRNA experiments have been carried out in cells cultured at optimal density to establish cell-cell adhesions. Thus, S100A11 knockdown allows genetic perturbation of E-cadherin-mediated cell-cell adhesion and recapitulates the plasma membrane and mitotic defects observed in sub-optimal cultures of mammary epithelial cells. Future experiments will be key to dissect these S100A11-mediated mechanisms to further understand how plasma membrane remodelling and cell-cell adhesions are coordinated during mitosis. Finally, as suggested by the Reviewer, we have now requalified our conclusions as appropriate in the revised manuscript.

- The current manuscript also demonstrates that cell adhesion is affected when S100A11 is knocked down (figure 4). It shows binding between and colocalization of S100A11 and E- cadherin, and shows that LGN cortical distribution is affected when S100A11 is knocked down (Figure 5). The results presented are suggestive of S100A11 being upstream of E-cadherin. However, I don't understand how the data shows "crosstalk between the plasma membrane, cell- cell adhesion, and the cell cortex during mitosis". For example, on P9: "We observed unequal distribution of CellMaskTM in a vast majority of S100A11-depleted cells (si-S100A11#1: ~79% versus si-Control: ~26%), indicating defects in plasma membrane remodelling (Figures 4B and 4C)." I don't agree that this demonstrates a defect in PM remodelling. Rather the cells in the representative images are less adherent and have adopted a more migratory cell state similar to that seen in figure 1 when seeded at low density. The fluidity of the much larger cells shown in knock down cells in panel F also appears higher, again suggesting an adhesion defect.

Authors response: The Reviewer has raised very important points here, which we would like to clarify.

We agree with the Reviewer that our results in S100A11-depleted cells indicate impaired cell adhesions which generates cells displaying an invasive/migratory behaviour. However, our analysis of S100A11-depleted mitotic cells labelled with CellMask^{TM} reveals abnormal plasma membrane elongation generating two daughter cells displaying defective geometry as compared to control cells. These defects in the plasma membrane and cell shape were not noticeable upon E-cadherin knockdown (see previous Figures 5K and 5L; now new Figures 6K and 6L). Thus, our results strongly suggest that S100A11 acts as an upstream cue that coordinates plasma membrane dynamics with E-cadherin-mediated cell adhesions, and that additional mechanisms may be regulated by S100A11 to coordinate cell-cell adhesion with plasma membrane remodelling. How S100A11 ensures such a dynamic interplay between the plasma membrane and E-cadherin during mitosis remains a key question that we have not fully addressed in this initial study. An attractive mechanism could be mediated by the function of S100A11 in regulating the dynamic interaction between F-actin and the plasma membrane, as previously reported (Jaiswal *et al.*, 2014 *Nat Commun*). Increasing evidence shows the importance of the crosstalk between the plasma

© 2023. Published by The Company of Biologists under the terms of the Creative Commons Attribution License (https://creativecommons.org/licenses/by/4.0/).

membrane, the cortex and cell shape for correct execution of mitosis (Rizzelli *et al.*, 2020 *Open Biol*). In our experiments, we show that impaired plasma membrane remodelling and cell shape are associated with defects in F-actin and astral microtubule organisation. Thus, our findings reinforce a model whereby S100A11 is a key membrane-associated protein that coordinates the crosstalk between the plasma membrane, cell-cell adhesion, and the cell cortex during mitosis. It will be key to characterise the interactome of S100A11 during mitosis to provide important mechanistic insights into this new role of S100A11; it is our intention to investigate this in the future.

To address the points raised by the Reviewer, we have changed and clarified the statements they highlighted above, in the revised manuscript (pages 10 and 11).

- *An earlier paper from the same lab this year identified Annexin A1 as directing mitotic spindle orientation via localising LGN at lateral cortex. During this earlier paper they also identified S100A11, which is a partner for Annexin A1. The authors could more clearly explain what S100A11 is in the current manuscript and how the current study builds on this earlier study.*

Authors response: We thank the Reviewer for highlighting our previous work characterising the interactome of LGN in mitotic mammary epithelial cells (Fankhaenel *et al.*, 2023 *Nat Comms*), and identifying Annexin A1 (ANXA1) as a polarity cue regulating the localisation and function of the evolutionarily conserved mitotic spindle orientation LGN complex. We also showed that ANXA1 direct partner S100A11 co-purifies with LGN and that perturbation of the ANXA1- S100A11 complex impairs the localisation of the LGN complex at the cell cortex during mitosis. Thus, as rightly pointed out by the Reviewer, this work builds on our previous work discussed above, but also on previous studies establishing S100A11 as a key regulator of plasma membrane repair by regulating the dynamic interplay between F-actin and the plasma membrane (Jaiswal *et al.*, 2014 *Nat Commun*), and studies showing that S100A11 interacts with E-cadherin at adherens junctions (Guo *et al.*, 2014 *Sci Signal*). To address the Reviewer's point (also raised by Reviewer 1), we have now included a paragraph in the introduction (page 5) and results (page 10) of the revised manuscript describing these and other functions of S100A11 to provide a strong rational to our decision to investigate this protein.

- *Based on the data presented, I suggest that the authors should requalify their data. I suggest that the conclusions that can be drawn from the data are that cellular state is important for regulating mitosis orientation and fidelity (i.e. adherent epithelia cells vs. less adherent more migratory cells). S100A11 is important for promoting cell-cell adhesions and might be upstream of the known role of E-cadherin in regulating spindle orientation. Whilst I suggest that more quantified experiments would need to be included in order to assess possible effects on plasma membrane remodelling, the manuscript could be generally improved by a clearer explanation of the open question that they are addressing and what specific advance this manuscript has made in relation to the current literature, including their own. I do not currently feel that the title of the manuscript is appropriate since I don't think that a crosstalk between the plasma membrane and cell-cell adhesion has been shown here.*

Authors response: We would like to reiterate our agreement with the Reviewer's suggestion about the conclusions drawn from our data. In the initial submission we proposed that perturbation of S100A11-mediated regulation of cell adhesion and plasma membrane impairs the identity of mammary epithelial cells, which affects their shape during mitosis leading to aberrant mitotic progression and outcome. While we have not checked the migratory behaviour of cells not forming cell-cell adhesions, we suggested that the cells adopted a mesenchymal phenotype. Furthermore, we discussed the implication of epithelial-to-mesenchymal transition on chromosome segregation fidelity and execution of mitosis, and how precisely they link with our study (see initial submission's pages 14 and 19). As suggested by the Reviewer, we have now clarified further these observations in the results (pages 7 and 11) and discussion (pages 15 and 19) of the revised manuscript.

We have quantified several aspects of the changes in plasma membrane dynamics and remodelling throughout, in the initial manuscript (Figure 1D-H; Figure 4C). To address the Reviewer's point, we have now added quantifications of membrane blebbing (new Figure 1I).

We would like to emphasise that the introduction of the initial manuscript has included the open questions that led to this study. These questions have been addressed further in the discussion, where we have also formulated new hypotheses and discussed what we think are the important

© 2023. Published by The Company of Biologists under the terms of the Creative Commons Attribution License (https://creativecommons.org/licenses/by/4.0/).

outstanding questions for future investigations, in light of our findings. In this study we demonstrate that maintaining epithelial identity is essential for correct execution of polarised cell divisions. Our findings indicate that mammary epithelial cells grown at sub-optimal density lose their epithelial identity, which results in several mitotic defects. We propose a novel mechanism in which S100A11 acts as a molecular sensor of external cues coordinating the interplay between plasma membrane dynamics and cell-cell adhesion to maintain epithelial identity and integrity, thereby ensuring correct progression, orientation, and outcome of cell division. As suggested by the Reviewer, we have clarified further the advances made in this study, in the revised Results and Discussion sections.

To address the Reviewer's final point, we would like to suggest the following revised title "Interplay between the plasma membrane and cell-cell adhesion maintains epithelial identity for correct polarised cell divisions", which we hope reflects better the results described in our studies.

Minor comments: important issues that can confidently be addressed.

- *P3: I wouldn't describe the junctional proteins listed as polarity proteins.*

Authors response: We have now made this rectification in page 3, as suggested by the Reviewer.

- *Figure 1 - can the membrane blebbing phenotype by quantified? At the moment this part is observational so can't really be used to determine the role of plasma membrane remodelling.*

Authors response: We have now included quantifications of blebbing in the revised manuscript, as suggested by the Reviewer (new Figure 1I).

- *Figure 3. I'm not sure what the 'subcortical actin cloud' measurement is. Figure 3G suggests it may be the distance from the cortex to the spindle pole but how does this relate to actin?*

Authors response: The Reviewer is right, the subcortical actin cloud includes a pool of dynamic subcortical actin that extends from the cortex (excluding the stiff cortical actin) to the cytoplasm, interacting with the centrosomes and concentrating near the retraction fibres. The subcortical actin cloud has been shown to mediate cortical forces and to concentrate force-generating proteins at the retraction fibres acting on centrosome dynamics and pulling on astral microtubules to orient the mitotic spindle (for example, please see Kwon *et al.*, 2015 *Dev Cell*). We have now included this clarification in the revised manuscript (page 10).

- *Figure 4A. I can't see GFP-S100A11 accumulating at the cell surface. To me these images suggest that it is relatively ubiquitously expressed throughout the cytoplasm and surface, which is different to the later antibody stains, that show localisation at the cell surface.*

Authors response: A similar point has been raised by Reviewer 1. Although our retroviral-mediated transduction allows to avoid excessive expression of GFP-S100A11, the ectopic S100A11 is expressed at higher levels as compared to its endogenous counterpart. Our live images show an accumulation of the protein at the cell surface, but relatively high levels are also visible in the cytoplasm (previous Figure 4A, new Figure S4A). By contrast immunolabelling for endogenous S100A11 shows an obvious accumulation of the protein at the plasma membrane. This difference could also be due to a dynamic behaviour of the protein translocation of GFP- S100A11 between the cell surface and cytoplasm that is captured in our live imaging. Similar slight differences between immunofluorescence and live imaging of cortical proteins involved in mitosis, such as Dynein, NuMA, LGN and CAPZB, have been reported in several studies (to cite a few: di Pietro *et al.*, 2017 *Curr Biol*; Elias *et al.*, 2014 *Stem Cell Rep;* Fankhaenel *et al.*, 2023). To address this point, we have now moved the panel showing S100A11 immunofluorescence in Figure S3A to new Figure 4A (also see response to Reviewer 1 Major Point 1).

- *Fig 4H doesn't show an active process of translocation of E-Cadherin to the cytoplasm. It shows representative images with slightly higher levels of E-Cadherin in the cytoplasm. This could be due to translocation or it could be to do with lack of E-Cadherin assembly.*

Authors response: We thank the Reviewer for pointing this out. We have rectified this statement

© 2023. Published by The Company of Biologists under the terms of the Creative Commons Attribution License (https://creativecommons.org/licenses/by/4.0/).

accordingly (page 11).

- *4I I don't understand where the line profile is derived from - where is apical and where is basal in the images? Could a diagram be included?*

Authors response: We have now included an illustration of this quantification, in the revised manuscript (new Figure 4J).

- *The discussion could be shortened and more clearly written - perhaps with subheadings of the main findings.*

Authors response: We have clarified several ideas and statements, based on the specific points addressed above. While it is challenging to reduce the size of this section, given that the study addresses several mechanisms of mitosis, we have now shortened the discussion in the revised manuscript.

- *Methods: Why is cholera toxin used in the cell culture medium?*

Authors response: Cholera toxin is a key component of MCF-10A medium, which has been shown to stimulate cAMP activation promoting cell proliferation in culture. This culture protocol is a gold standard in the field (Debnath *et al.*, 2023 *Methods*). Given that cholera toxin is a highly regulated chemical and takes several months to purchase, we have tried culturing MCF-10A without the toxin, but this negatively affected proliferation and passage of this cells. Therefore, we concluded that adding it to the culture medium is important.

Reviewer #3 (Significance (Required)):

In general, this is an interesting paper about the fidelity of mitosis in cells in adherent monolayers vs. in more migratory, non-adherent states. There is existing literature on this topic (some cited in the manuscript, alongside reviews of the topic).

The main conceptual advance, as far as I can see, is that S100A11 is important for promoting cell-cell adhesions and might be upstream of the known role of E-cadherin in regulating spindle orientation via LGN. The main limitation is that plating cells at different densities is not a direct 'perturbation' of cell-cell adhesion. This means that the phenotypes seen could be due to many factors, not just cell adhesion. Assessment of plasma membrane and cytoskeletal dynamics are also often observational and not conclusive.

The manuscript would be of interest to basic researchers working on epithelial development. Also potentially to basic researchers working on cancer, due to the mitotic errors described.

I have expertise in epithelial cell biology.
I estimate the authors would need between 3 and 6 months for revisions if they decide to do further experiments and between 1 and 3 months if they decide to re-qualify their claims.

Authors response: We thanks the Reviewer for their overall positive feedback on our work and its broader importance for researchers in epithelial development and cancer biology.

We would like to reiterate our agreement with the Reviewer's assessment of the conceptual advances of our work. We show that S100A11 complexes with E-cadherin and LGN during mitosis to control cell-cell adhesion assembly and the mitotic spindle machinery, respectively, which in turn ensures faithful chromosome segregation. Our results also suggest that S100A11 lies upstream of E-cadherin in the regulation of the LGN-mediated mitotic spindle machinery. We also agree with the Reviewer that plating epithelial cells at low density does not directly affect cell-cell adhesion, because, in these culture conditions, cells are not dense enough to establish cell-cell contacts necessary to assemble stable adherens junctions. Rather, and as rightly pointed out by the Reviewer, cells grown at low density fail to maintain their epithelial identity and adopt a more mesenchymal and elongated behaviour, which is accompanied by dramatic changes in plasma membrane remodelling throughout mitosis. Interestingly, our results show that both S100A11 and E-cadherin do not localise at the plasma membrane in these sub-optimal culture conditions. This

© 2023. Published by The Company of Biologists under the terms of the Creative Commons Attribution License (https://creativecommons.org/licenses/by/4.0/).

along with our results showing that depletion of S100A11 phenocopies the effect of low-density culture conditions on plasma membrane remodelling and E-cadherin mediated cell-cell adhesion assembly, allow us to propose a mechanism whereby the membrane-associated S100A11 protein acts as a molecular sensor of external cues bridging plasma membrane remodelling to E- cadherin-dependent cell adhesion to coordinate correct progression and outcome of mammary epithelial cell divisions.

We are grateful for the Reviewer's insightful discussion of our findings. As we discussed above in our responses to their specific points, we have requalified many of our statements to clarify further our main findings and conclusions throughout the revised manuscript. We have also added new quantifications in response to the Reviewer's suggestions. We believe, that together, these revisions have advanced further the initial manuscript.

Original submission

First decision letter

MS ID#: JOCES/2023/261701

MS TITLE: Interplay between the plasma membrane and cell-cell adhesion maintains epithelial identity for correct polarised cell divisions

AUTHORS: Manal M Hosawi, Jiaoqi Cheng, Maria Fankhaenel, Marcin R Przewloka, and Salah Elias
ARTICLE TYPE: Research Article

I am happy to tell you that your manuscript transferred from ReviewCommons has been accepted for publication in Journal of Cell Science, pending standard ethics checks. I felt that your response to Reviewers 1 and 2 in the ReviewCommons response adequately address their concerns. I returned the manuscript to previous Reviewer 3, who indicated that they are also happy in principle with your response. This referee reports is appended below. The reviewer does mention two minor points which you may like tohighlight and clarify in the text. If you do wish to do so, please respond to this email with the updated text.

Reviewer 1

Advance summary and potential significance to field

This is an interesting paper about the fidelity of mitosis in cells in adherent monolayers vs. in more migratory, non-adherent states. The main conceptual advance is that S100A11 is important for promoting cell-cell adhesions and might be upstream of the known role of E-cadherin in regulating spindle orientation via LGN. The manuscript would be of interest to basic researchers working on epithelial development. Also potentially to basic researchers working on cancer due to the mitotic errors described.

Comments for the author

Thank you to the authors for their revisions.
The further background information for S100A11 is very helpful.
Together with the requalification of text and additional summaries, the manuscript is now much clearer. Figure 7 is also a helpful summary. There are a couple of points that I suggest could still be made clearer, outline below. However, I suggest that the manuscript be accepted for publication.

As the authors point out in their rebuttal, the uniform distribution of cell mask around the membrane in Ecad siRNA when compared to unequal distribution following S100A11 siRNA (e.g. Fig 4C) is important, since this demonstrates that an adhesion defect does not solely explain this unequal membrane distribution. I suggest that this point could be highlighted in the text, perhaps with a direct comparison of cellmask in Ecad siRNA cells.

© 2023. Published by The Company of Biologists under the terms of the Creative Commons Attribution License (https://creativecommons.org/licenses/by/4.0/).

Figure 3F&G. The extra information about the definition of a subcortical actin cloud is nice but my comment on this figure was that it’s not clear what was measured to quantify the depth of the subcortical actin cloud. The diagram in G suggests that the measurement may be the distance from the cortex to the spindle pole but that may not be coincident with the extent of the subcortical actin cloud.

© 2023. Published by The Company of Biologists under the terms of the Creative Commons Attribution License (https://creativecommons.org/licenses/by/4.0/).
